# Single-Cell Virus Sequencing of Influenza Infections That Trigger Innate Immunity

**DOI:** 10.1128/JVI.00500-19

**Published:** 2019-06-28

**Authors:** Alistair B. Russell, Elizaveta Elshina, Jacob R. Kowalsky, Aartjan J. W. te Velthuis, Jesse D. Bloom

**Affiliations:** a Basic Sciences and Computational Biology, Fred Hutchinson Cancer Research Center, Seattle, Washington, USA; b Division of Virology, Department of Pathology, University of Cambridge, Cambridge, United Kingdom; c Department of Genome Sciences, University of Washington, Seattle, Washington, USA; d Howard Hughes Medical Institute, Seattle, Washington, USA; University of Texas Southwestern Medical Center

**Keywords:** 10x Chromium, NS1, PB1, PacBio, defective virus, heterogeneity, influenza virus, interferon, single-cell RNA-seq

## Abstract

Influenza virus-infected cells vary widely in their expression of viral genes and only occasionally activate innate immunity. Here, we develop a new method to assess how the genetic variation in viral populations contributes to this heterogeneity. We do this by determining the transcriptome and full-length sequences of all viral genes in single cells infected with a nominally “pure” stock of influenza virus. Most cells are infected by virions with defects, some of which increase the frequency of innate-immune activation. These immunostimulatory defects are diverse and include mutations that perturb the function of the viral polymerase protein PB1, large internal deletions in viral genes, and failure to express the virus’s interferon antagonist NS1. However, immune activation remains stochastic in cells infected by virions with these defects and occasionally is triggered even by virions that express unmutated copies of all genes. Our work shows that the diverse spectrum of defects in influenza virus populations contributes to—but does not completely explain—the heterogeneity in viral gene expression and immune activation in single infected cells.

**IMPORTANCE** Because influenza virus has a high mutation rate, many cells are infected by mutated virions. But so far, it has been impossible to fully characterize the sequence of the virion infecting any given cell, since conventional techniques such as flow cytometry and single-cell transcriptome sequencing (scRNA-seq) only detect if a protein or transcript is present, not its sequence. Here we develop a new approach that uses long-read PacBio sequencing to determine the sequences of virions infecting single cells. We show that viral genetic variation explains some but not all of the cell-to-cell variability in viral gene expression and innate immune induction. Overall, our study provides the first complete picture of how viral mutations affect the course of infection in single cells.

## Abstract (precis)

Because influenza virus has a high mutation rate, many cells are infected by mutated virions. But so far, it has been impossible to fully characterize the sequence of the virion infecting any given cell, since conventional techniques such as flow cytometry and single-cell transcriptome sequencing (scRNA-seq) only detect if a protein or transcript is present, not its sequence. Here we develop a new approach that uses long-read PacBio sequencing to determine the sequences of virions infecting single cells. We show that viral genetic variation explains some but not all of the cell-to-cell variability in viral gene expression and innate immune induction. Overall, our study provides the first complete picture of how viral mutations affect the course of infection in single cells.

## INTRODUCTION

Infection with an acute virus such as influenza initiates a race between the virus and the immune system. As the virus spreads, some cells detect infection and begin producing interferon (IFN). This IFN directs expression of antiviral interferon-stimulated genes (ISGs) in the infected cell and its neighbors via autocrine and paracrine signaling, as well as helps launch a broader immune response (1, 2). If innate immunity is activated sufficiently rapidly, it can reduce viral replication and disease (3–7); although, excessive immune responses later in infection can actually be associated with immunopathology and severe disease (8, 9).

Unfortunately for the host, the influenza virus initially only rarely triggers IFN production by infected cells (10, 11). This rareness of IFN induction is just one form of the extreme cell-to-cell heterogeneity that characterizes infection: cells also vary widely in their production of viral mRNA, proteins, and progeny virions (12–16). Because viral growth and the IFN response both amplify themselves, early variation in the initiation of these events could have significant downstream consequences for the race between virus and immune system, especially since natural human infections are typically founded by just a few virions entering a few cells (17–19).

It is unclear why only some infected cells trigger innate immune responses. Two possible contributors are pure stochasticity and preexisting variation in cellular state. For instance, only some cells induce IFN even upon treatment with synthetic innate immune ligands (20–22), and the frequency of IFN induction may depend on a cell’s preexisting chromatin state (23). But for influenza virus, a third possible contributor also looms large: viral genetic diversity. The virus has evolved mechanisms to avoid IFN induction, including expressing proteins that interfere with innate immune induction (24–28) and sequestering immunogenic viral RNA (29). However, because influenza virus has a high mutation rate (30–34), individual virions often have genetic defects that could impair these immune evasion strategies. Indeed, many studies have identified mutations that increase IFN induction when engineered into a viral population (11, 35–37), and viral stocks that are rich in internal deletions in the polymerase genes induce more IFN (16, 38–42).

However, existing techniques are inadequate to determine how viral genetic diversity contributes to cell-to-cell heterogeneity during infection. Flow cytometry and fluorescent reporters only measure protein levels (14, 43, 44), and current single-cell transcriptomic techniques measure abundance of transcripts and provide only fragmentary information on their sequences (12, 13, 16, 45–48). None of these techniques reliably reveal if the virion infecting a specific cell has some idiosyncratic mutation.

Here, we developed a new approach to measure both the full transcriptome and sequences of all viral genes in single influenza virus-infected cells. To do this, we performed both standard Illumina-based transcriptomics and full-length PacBio sequencing of viral genes from single cells. Two-thirds of cells were infected by virions that had a mutation or defect in gene expression. We identified several types of viral defects that increased IFN induction, but also showed that viral diversity was insufficient to fully explain cell-to-cell heterogeneity during influenza virus infection.

(This article was submitted to an online preprint archive [49].)

## RESULTS

### A system to identify and enrich rare IFN^+^ cells.

**FIG 1:**
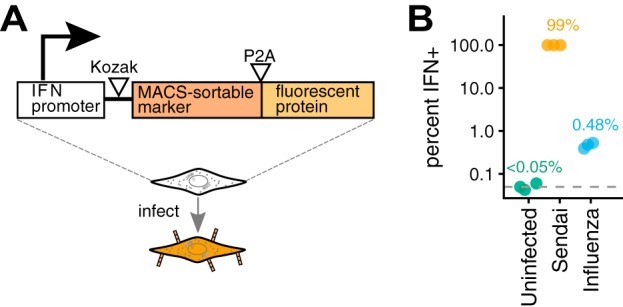
Reporter cells to identify and enrich infections that activate IFN expression. (A) The reporter consists of an IFN promoter that drives expression of a cell surface protein amenable to MACS and a fluorescent protein. We created reporters with type I and type III IFN promoters (see File S1 in the supplemental material). In A549 cells, the reporters were efficiently activated by an IFN-inducing strain of Sendai virus (Fig. S1A). (B) Frequency of IFN induction upon infection with the influenza virus stock used in the single-cell studies in this paper, as quantified using the type III IFN reporter (see Fig. S2 for full flow cytometry data). The plot also shows data from uninfected cells and cells infected with Sendai virus. The limit of detection of 0.05% is indicated with a dashed line, and numbers show the medians from three measurements.

Influenza virus only rarely triggers IFN expression in infected cells (10–12)—a fact that poses a challenge for the study of IFN induction in single cells. Therefore, we developed a method to identify and enrich rare IFN^+^ cells by creating A549 cells that carried IFN reporters consisting of a type I (*IFNB1*) or type III (*IFNL1*) promoter driving expression of a cell-surface protein (LNGFRΔC [50, 51]) followed by a fluorescent protein (Fig. 1A). Cells that activate the IFN reporters can be enriched by magnetic-activated cell sorting (MACS) or identified by flow cytometry. The reporters were efficiently activated by infection with a strain of Sendai virus (52) that potently induces IFN (see Fig. S1A in the supplemental material), and activation of the type I and type III IFN reporters was highly correlated in our cells (Fig. S1B; further validated by the single-cell transcriptomics below). Therefore, for the rest of this paper, we use “IFN expression” to refer to combined expression of type I and III IFNs.

We generated a stock of A/WSN/1933 (H1N1) influenza virus (here referred to as WSN) directly from reverse genetics plasmids (53), and passaged this stock at a low multiplicity of infection (MOI). This process ensures that the viral stock is relatively “pure,” with only low levels of the large internal deletions and other defects that arise in stocks passaged at a high MOI (54). As described in the next subsection, our stock actually consisted of a mix of two viruses: wild-type WSN and a variant of this virus that carries synonymous viral “barcodes” near the termini of each gene. This viral stock activated the IFN reporter in ∼0.5% of infected cells (Fig. 1B), a frequency roughly comparable to that reported in prior studies (11, 12). We also validated that MACS for the cell surface protein driven by the IFN reporter enriched the IFN^+^ cells by >50-fold (see Fig. S3).

### Combined transcriptomics and virus sequencing of single infected cells.

**FIG 2:**
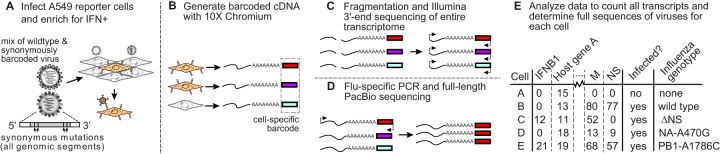
Approach for combined transcriptomics and viral sequencing of single influenza virus-infected cells that express IFN. (A) IFN reporter A549 cells are infected with a mix of wild-type and synonymously barcoded viruses. IFN^+^ cells are enriched by MACS and pooled with nonenriched cells and uninfected canine cells that serve as controls for multiplets and mRNA leakage. (B) The mRNAs from individual cells are converted to cDNAs tagged with cell-specific barcodes. (C) Cellular transcriptomes are quantified using standard single-cell 3′-end Illumina sequencing, and (D) viral genes are enriched by influenza virus-specific PCR and fully sequenced by PacBio (in this schematic, only the cell labeled by the red barcode is infected and has viral transcripts that are sequenced by PacBio). (E) The result is a matrix giving the expression of each gene in each cell, as well as the full sequences of the viral genes in infected cells.

We developed the approach shown in Fig. 2 to obtain the entire transcriptome and the full sequences of all viral genes in single cells. First, we generated the viral stock described in the previous subsection, which consisted of a mix of wild-type WSN and a “synonymously barcoded” variant that contained two engineered synonymous mutations near each termini of each gene (see File S2). These viral barcodes allow us to identify coinfections from single-cell transcriptomic data (12) and provide a control for PCR artifacts during full-length sequencing of viral transcripts (see below). We used this viral stock to infect A549 IFN reporter cells (Fig. 2A) at a dose that led to detectable viral transcription in ∼25% of cells (this moderately low MOI reasonably balances our desire to limit the number of coinfections with the cost of performing transcriptomics on uninfected cells). From 12 to 13 h postinfection, we used MACS to enrich cells that activated the IFN reporter. To ensure the presence of IFN-negative cells, we added back nonenriched cells to ∼10% of the total. We also added uninfected canine cells to ∼5% of the total as a control for multiplets and to estimate the background amount of viral mRNA detected in truly uninfected cells.

We processed the cells on a commercially available platform (55) that isolates cells in droplets and reverse transcribes polyadenylated mRNAs to append a unique cell barcode to all cDNAs in each droplet and a unique molecular identifier (UMI) to each cDNA molecule (Fig. 2B). Because influenza virus mRNAs are polyadenylated (56), this process appends cell barcodes to viral as well as cellular mRNAs. Furthermore, because virtually the entire influenza virus genome is transcribed, the cell-barcoded cDNA spans almost all 13,581 nucleotides in the segmented viral genome: the only portions not covered are one universally conserved nucleotide upstream of the transcription start site (57) and 17 to 22 highly conserved nucleotides downstream of the polyadenylation site (56) in each of the eight viral gene segments.

We used a portion of the cell-barcoded cDNA for standard single-cell transcriptomics by Illumina 3′-end sequencing (Fig. 2C), but we also took a portion and enriched for full-length viral molecules by PCR (Fig. 2D). We performed PacBio sequencing on these full-length viral cDNAs to generate high-accuracy circular consensus sequences (CCSs) (58). These CCSs retain the cell barcodes, and with sufficient sequencing depth, we obtained CCSs from multiple unique UMI-tagged cDNAs for each viral gene in each cell. Because most cells were infected by just one or two virions, we were able to build a consensus of CCSs for each viral gene in each cell to determine the sequence(s) of these virions. Combining this information with the 3′-end sequencing determined the entire transcriptome and the full sequences of the infecting virions in single cells (Fig. 2E).

### Transcriptomic analyses of single IFN^+^ and IFN^−^ influenza virus-infected cells.

**FIG 3:**
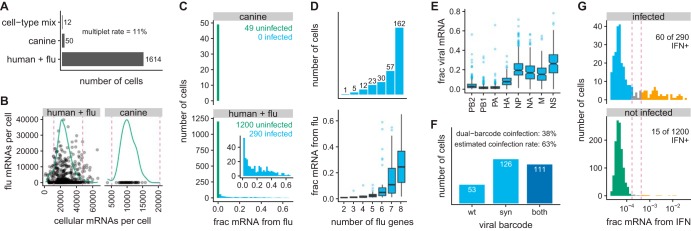
Single-cell transcriptomics of IFN-enriched influenza virus-infected cells. (A) Numbers of cells from which transcriptomes were obtained. From these numbers, we estimate (59) that approximately 11% of the transcriptomes were derived from multiple cells. (B) The numbers of cellular and viral mRNAs detected for each cell are plotted as a point. Green lines show the distribution of cellular mRNAs per cell. Cells outside the dashed magenta lines have unusually low or high cellular mRNA (likely low-quality emulsions or multiplets) and were excluded from subsequent analyses. (C) Distribution across cells of the fraction of all mRNA derived from influenza virus. Cells called as infected are in blue, while other cells are in green. The inset shows the amount of viral mRNA in the human cells that are called as infected. (D) Numbers of influenza genes detected per infected cell, and the amounts of viral mRNA in cells expressing each number of viral genes. Figure S4 shows the frequency that each viral gene is detected. (E) Relative expression of viral genes, quantified as the fraction of all viral mRNA in each infected cell derived from each gene. (F) Numbers of cells infected with wild-type virus, synonymously barcoded virus, or both. From the cells infected with both viral barcodes, we estimate (59) that 63% of infected cells were coinfected. (G) Fractions of cellular mRNA from IFN across cells, faceted by whether the cells were infected. Cells to the left of the first dashed magenta line were classified as IFN^−^ and cells to the right of the second line as IFN^+^. A pseudocount is added to the number of IFN transcripts detected in each cell, which is why none of the fractions are zero.

We obtained transcriptomes for 1,614 human (A549) cells and 50 of the uninfected canine cells that were spiked into the experiment as a control (Fig. 3A). We also obtained 12 transcriptomes with a mix of human and canine transcripts; from the number of such mixed cell-type transcriptomes, we estimate (59) that ∼11% of the transcriptomes were derived from multiple cells. To remove some of these multiplets along with low-quality droplets, we filtered transcriptomes with unusually high or low numbers of cellular transcripts as is commonly done in analysis of single-cell transcriptome sequencing (scRNA-seq) data (60). After this filtering, we retained 1,490 human cells for further analysis (Fig. 3B).

To identify infected cells, we examined the fraction of each transcriptome derived from virus (Fig. 3C). As expected, only a small fraction (∼0.7%) of transcripts in the uninfected canine cells was viral; this low-level background was likely from lysed cells that released ambient viral mRNA. We tested whether each cell contained significantly more viral transcripts than expected under a Poisson model given this background fraction and classified 290 human cells as definitively infected with influenza virus (Fig. 3C). We classified the other cells as uninfected, although it is possible that some were infected with virions that produced very little mRNA. The distribution of the amounts of viral mRNA across infected cells is shown in the inset in Fig. 3C. As in our prior work (12), the distribution is extremely heterogeneous: many infected cells had only a few percent mRNA derived from virus, but viral mRNA comprised more than half the transcriptome of a few cells.

We called the presence or absence of each viral gene in each infected cell, again using a Poisson model parameterized by background fractions estimated from uninfected canine cells. We called presence/absence of genes rather than transcripts, since the two genes that encode multiple transcripts (M1/M2 from the M gene, and NS1/NS2 from the NS gene) do so via alternative splicing that leaves both isoforms with the same termini, making them indistinguishable by 3′-end sequencing. Figure 3D (top) shows that 162 of 290 infected cells expressed all eight genes (see Fig. S4 for frequencies of individual genes). This measured frequency of infected cells expressing all eight genes is slightly higher than in our own prior work using the WSN strain (12) and slightly to substantially higher than that reported in studies by others using different viral strains or methodologies (15, 43, 61, 62).

The amount of viral mRNA was lower in cells that failed to express viral genes (Fig. 3D, bottom). However, viral burden remained highly variable even after conditioning on the number of viral genes: some cells that failed to express one or even two genes still derived >50% of their mRNA from virus, while other cells that expressed all genes had only a few percent mRNA from the virus (Fig. 3D, bottom). Consistent with our prior work (12), despite the wide variation in absolute expression of viral genes, their relative expression was fairly consistent (Fig. 3E) and generally matched values from older bulk studies (63).

By examining the synonymous viral barcodes near the 3′ termini of transcripts, we determined that 38% of cells were coinfected with wild-type and synonymously barcoded virions (Fig. 3F; cells called coinfected if a binomial test rejected the null hypothesis that 95% of viral mRNA is from one viral barcode variant). From Fig. 3F, we estimate (59) that 63% of infected cells were coinfected. Interestingly, this coinfection rate is higher than expected from the relative numbers of infected and uninfected cells (Fig. 3C) if infection was Poisson. This discrepancy could arise if the MACS for IFN^+^ cells also enriched coinfected cells, if infection was not truly Poisson, or if coinfection complemented otherwise transcriptionally defective virions to increase the likelihood that we identify a cell as infected. The first explanation seems unlikely, as there was no tendency for coinfected cells to express more IFN (see Fig. S5). Therefore, we favor the latter two explanations, both of which have been demonstrated for other viruses (64, 65). The moderately high rate of coinfection may also explain why more cells in our experiment expressed all eight viral genes compared to those in some prior studies, as a coinfecting virion can complement a missing viral gene.

We next examined expression of IFN and ISGs (Fig. 3G and Fig. S6). More than 20% of infected cells were IFN^+^, indicating that the MACS successfully enriched IFN^+^ cells far beyond their initial frequency. The expression of type I and type III IFN was highly correlated in single cells, justifying our decision to collapse both classes under the single label of “IFN” in the analyses that followed (see Fig. S7). Few (∼1.3%) uninfected cells were IFN^+^; the few that were present might be because the MACS enriched for rare cells that activated IFN in response to nonviral ligands (66–68) or because some cells that we classified as uninfected were actually infected at low levels. The difference in the frequency of IFN positivity among infected and uninfected cells in Fig. 3G was highly significant (*P* < 10*^−^*^5^, Fisher’s exact test). Many more cells expressed ISGs than IFN itself (Fig. S6A). The IFN^+^ cells were a subset of the ISG^+^ cells: IFN^+^ cells always expressed ISGs, but many ISG^+^ cells did not express IFN (Fig. S6B). These results are consistent with the established knowledge that IFN is expressed primarily in cells that directly detect infection but that ISGs are also expressed via paracrine signaling in other cells (1, 2).

Finally, we qualitatively examined how expression of viral genes, IFN, and ISGs relates to the overall structure of the high-dimensional transcriptomic data. Figure S8 shows unsupervised t-SNE clustering (69) of the cells. Cells expressing high levels of viral genes, IFN, and ISGs clustered together, and most of the structure in the t-SNE plot that is not associated with these genes involves uninfected and IFN^−^ cells.

### Full genotypes of viruses infecting single IFN^+^ and IFN^−^ cells.

We next used PacBio sequencing (Fig. 2D; see also File S3) to determine the full sequences of the viral genes in single infected cells. We obtained >200,000 high-quality PacBio CCSs that mapped to an influenza virus gene and contained a cell barcode and UMI (see Fig. S9). The synonymous viral barcodes at both termini of each gene enabled us to confirm that PCR strand exchange was rare (see Fig. S10), meaning that the vast majority of CCSs correctly link the sequence of the transcript to cell barcodes and UMIs that identify the cell and molecule of origin.

After calling the presence/absence of each viral gene in each cell as described in the previous section, we called mutations if they were found in at least two CCSs originating from different mRNAs (unique UMIs) and at least 30% of all CCSs for that gene in that cell. For cells coinfected with both viral barcode variants, we called mutations separately for each viral variant. This strategy reliably identifies mutations in virions that initiate infection of cells infected with at most one virion of each viral barcode variant (∼75% of infected cells) as well as high-abundance mutations in cells coinfected with multiple virions of the same viral barcode. It will not identify mutations that arise within a cell after the first few rounds of viral genome replication, since such mutations will not reach 30% frequency in that cell. Therefore, analogous to somatic variant calling in tumor sequencing (70, 71), there is a limit to our detection threshold: we cannot identify mutations that occur in just a small fraction of transcripts in a cell.

We were able to call the sequences of all expressed viral genes in the majority of infected cells (see Fig. S11). We were most effective at calling full viral genotypes in cells that expressed large amounts of viral mRNA and were infected by only one viral barcode variant (Fig. S11), but we also called full genotypes for many cells that had low viral burden or were coinfected by both viral barcode variants.

**FIG 4:**
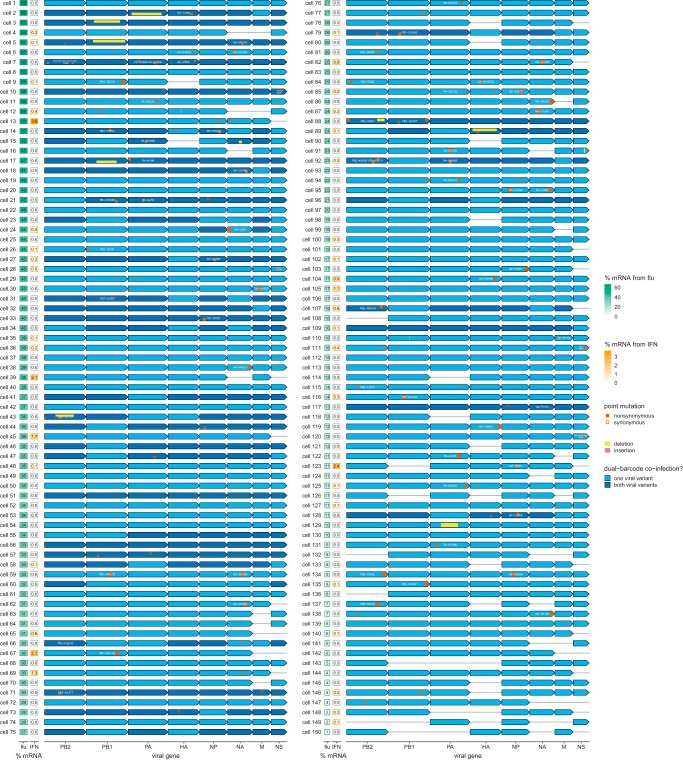
Viral genotypes and infection outcomes in single cells. Green and orange boxes at the left show the percentage of all mRNA in that cell derived from virus and the percentage of all cellular mRNA derived from IFN, respectively. The second box is framed in orange for cells classified as IFN^+^ in Fig. 3G. Blue arrows indicate the presence of a viral gene from one (light blue) or both (dark blue) viral barcode variants; a dark blue arrow therefore means that a cell was coinfected. Circles and boxes on the arrows indicate mutations or indels as described in the legend at right. The circle areas and box heights are proportional to the fraction of CCSs with that mutation. For dual-barcode infections, mutations/indels for the wild-type and synonymously barcoded viral variants are shown in the top and bottom half of the arrows, respectively. For instance, cell 5 was coinfected by a virion with one unmutated and one internally deleted copy of PB1.

The 150 cells for which we called the full viral genotypes are shown in Fig. 4 (see also File S4). Visual inspection of this figure reveals a wealth of information. For instance, the cell with the highest viral burden (cell 1 in Fig. 4, which has 65% of its mRNA from virus) was infected by a virion that expressed unmutated copies of all eight genes and did not induce detectable IFN. However, 12 of the other 13 cells with at least 50% of their mRNA from virus were infected by virions that had a mutation or failed to express a gene, and five of these cells produced IFN. As expected, all cells infected by virions that failed to express a component of the viral polymerase complex (PB2, PB1, PA, or NP) expressed small amounts of viral mRNA, since they were limited to primary transcription using incoming proteins (e.g., cell 132 and cell 143). The two cells that expressed the most IFN (cell 13 and cell 123) lacked the viral NS gene that encodes the virus’s primary IFN antagonist (24, 25). Many other IFN^+^ cells had different defects, such as large internal deletions (e.g., cell 5 and cell 89) or amino acid mutations (e.g., cell 9, cell 28, and many others).

However, Fig. 4 also reveals stochasticity that is independent of viral genotype. This stochasticity sometimes acts to the detriment of the virus and sometimes to the detriment of the cell. As an example of the former case, expressing unmutated copies of all eight genes did not guarantee high viral gene expression and successful innate immune evasion: for instance, the unmutated virion that infected cell 139 only managed to express viral mRNA to 6% of the total transcriptome, and the unmutated virion that infected cell 105 still induced IFN. But in other cases, the stochasticity allows a defective virus to still escape immune recognition. For instance, there are a number of cells (e.g., cell 62 and cell 78) that did not activate IFN despite being infected by virions that failed to express NS.

### Viral defects associated with viral gene expression and IFN induction in single cells.

**FIG 5:**
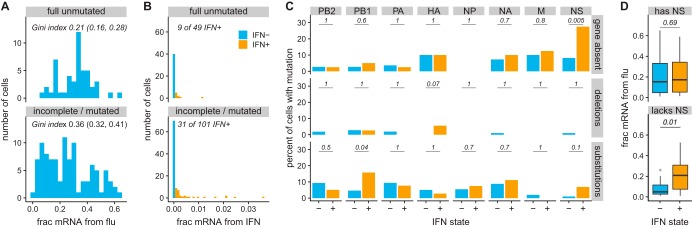
Viral features associated with heterogeneity in infection outcome among cells for which we determined viral genotypes. (A) Percentages of all mRNA derived from virus, faceted by whether cells expressed unmutated copies of all eight genes. Cells infected by fully unmutated virions exhibited less heterogeneity in viral burden as quantified by the Gini index (95% confidence intervals are indicated). (B) IFN expression among cells expressing unmutated copies of all genes and among cells with mutations or missing genes. (C) Specific viral defects associated with IFN induction. The top panel shows the percentages of IFN^−^ and IFN^+^ cells that failed to express each viral gene. The middle and bottom panels show the percentages of IFN^−^ and IFN^+^ cells that had a deletion or amino acid substitution in each gene, conditioned on the cell expressing that gene. Numbers give *P* values (Fisher’s exact test) for rejecting the null hypothesis that percentages are equal among IFN^−^ and IFN^+^ cells. (D) There was no association between IFN induction and the amount of viral mRNA in cells that expressed NS, but viral burden was associated with IFN induction among cells that lacked NS. Throughout this figure, we only consider substitutions that are nonsynonymous.

To systematically assess viral features associated with infection outcome, we divided the 150 cells in Fig. 4 into those that expressed unmutated copies of all eight genes (disregarding synonymous mutations) and those that did not. Figure 5A shows that the 49 cells infected by full unmutated virions had a significantly tighter distribution of the amount of viral mRNA per cell than the other 101 cells as quantified by the Gini index (72) (see also File S5). Therefore, viral defects are a major contributor to the heterogeneity in viral transcriptional burden.

Some viral defects also contribute to IFN induction. Specifically, cells infected by incomplete or mutated virions expressed IFN more frequently than cells infected by virions that expressed unmutated copies of all genes (Fig. 5B), although this difference was not statistically significant (*P* = 0.12, Fisher’s exact test). However, the association was significant for certain classes of viral defects: absence of NS and amino acid mutations in PB1 were significantly enriched in IFN^+^ cells, and amino acid mutations in NS and deletions in HA were weakly enriched (Fig. 5C). The only trend that remained significant at a false discovery rate (FDR) of 10% was absence of NS. This lack of statistical significance after FDR correction could be due to the relatively modest number of fully sequenced infected cells (just 150). The validation experiments in the next section show that many of the viral mutations in IFN^+^ cells do in fact increase the rate of IFN induction.

One other interesting trend emerged from the single-cell data. There was no difference in the amounts of viral mRNA between IFN^+^ and IFN^−^ cells that expressed NS (Fig. 5D). But among cells that lack NS, cells with more viral mRNA were significantly more likely to be IFN^+^ (Fig. 5D); this finding is elaborated on in the validation experiments below. Overall, the lack of reduced viral gene expression in IFN^+^ cells suggests that autocrine IFN signaling typically occurs too late to suppress viral transcription, and the well-known inhibitory effect of IFN against influenza virus depends mainly on paracrine signaling.

### Validation that viral defects in single IFN^+^ cells often increase IFN induction.

To test if the viral defects identified in single IFN^+^ cells caused increased IFN expression, we used reverse genetics to generate bulk stocks of viruses with some of these defects.

The viral defect most strongly associated with IFN induction was failure to express the NS gene (Fig. 4 and 5C). Although it is sometimes possible to use complementing cells to generate influenza viruses lacking a specific gene (73, 74), we were unable to generate viruses that lacked NS. The NS gene encodes two proteins (NS1 and NS2), the first of which is influenza’s primary innate immune antagonist (24, 25). We therefore mimicked the absence of NS by creating a mutant virus (which we term “NS1stop”) that had multiple stop codons early in the NS1 coding sequence.

The single-cell data also showed that amino acid substitutions in proteins encoded by the PB1 and NS genes were enriched in IFN^+^ cells (Fig. 4 and 5C), and so we created mutant viruses with some of these substitutions: PB1-D27N, PB1-G206S, PB1-K279R, PB1-T677A, NS1-A122V, and NS2-E47G.

Finally, prior work has suggested that virions with internal deletions in the polymerase genes can induce higher levels of IFN (16, 38–42). Although such deletions are not significantly enriched among IFN^+^ cells in our single-cell data (Fig. 5C), there was a coinfected IFN^+^ cell where one viral variant had a deletion in PB1 spanning nucleotides 385 to 2163 (cell 5 in Fig. 4). We therefore created a virus carrying this deletion and propagated it in cells constitutively expressing PB1 protein.

**FIG 6:**
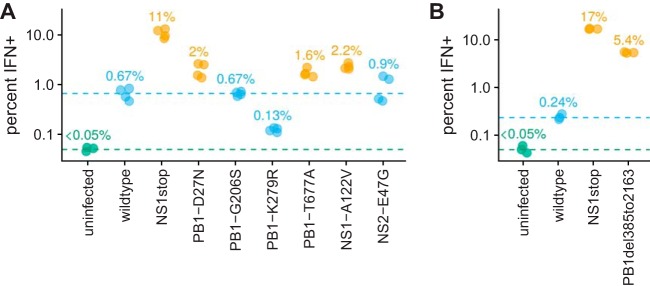
Validation that IFN induction is increased by some of the mutations identified in the single-cell virus sequencing of IFN^+^ cells. (A) Percentages of infected cells that became IFN^+^ after infection with a bulk stock of the indicated viral mutant, as determined using a reporter cell line. The numbers indicate the medians from four measurements for each viral mutant. The limit of detection of 0.05% is indicated with a dashed green line, and the median value for the wild-type viral stock is indicated with a dashed blue line. Points are colored orange if the mutant virus stock induced IFN more frequently than the wild-type viral stock (one-sided *t* test, *P* < 0.01) and blue otherwise. (B) Similar to the first panel but validates increased IFN induction for a large internal deletion in the PB1 gene and normalizes infecting virion dose rather than calling IFN^+^ percentage only among infected cells. See Fig. S12 and S13 for details. The experiments in the two panels were performed on different days, and so numerical values can be reliably compared within panels but not between panels.

We tested the rate of IFN induction by each viral stock using the reporter cells. Figure 6 shows that five of the eight mutant viral stocks induced IFN more frequently than a wild-type viral stock. The strongest IFN induction was by the NS1stop virus, but the PB1 internal deletion and three of the point mutant viruses (PB1-D27N, PB1-T677A, and NS1-A122V) also induced IFN significantly more frequently than the wild type. The other three point mutants (PB1-G206S, PB1-K279R, and NS2-E47G) did not increase IFN induction, an unsurprising finding since we expected some mutations without an IFN-enhancing effect to be found in IFN^+^ cells by chance. Overall, the results in Fig. 6 validate that the viral defects in single IFN^+^ cells often cause increased IFN production.

However, IFN induction remained stochastic even for the most potently IFN-inducing viral mutants. Figure 6 shows flow cytometry data (see also Fig. S12), which is itself a single-cell measurement, albeit one that does not report the viral genotype. As can be seen from these data, none of the mutant viral stocks induced IFN in more than 20% of infected cells. Of course, these mutant virus stocks are themselves genetically heterogeneous, as many virions will have additional defects similar to that revealed by our single-cell sequencing of the “wild-type” viral stock. However, our single-cell data show that IFN induction was stochastic even for infections that shared the same defect, such as absence of NS (e.g., compare cell 62 and cell 69 in Fig. 4). Therefore, the experiments in this section not only validate some specific viral defects that increase IFN induction but also show that induction remains stochastic even with these defects.

### The IFN-inducing viral defects act by diverse mechanisms.

Some of the viral defects in IFN^+^ cells are easy to reconcile with existing knowledge: for instance, NS1 is the virus’s primary IFN antagonist (24, 25), and internal deletions are prevalent in immunostimulatory viral stocks (16, 38–42). Other defects are more surprising: for instance, it is not obvious why amino acid mutations in PB1 increase IFN induction. We therefore designed experiments to interrogate some of these defects in more detail.

**FIG 7:**
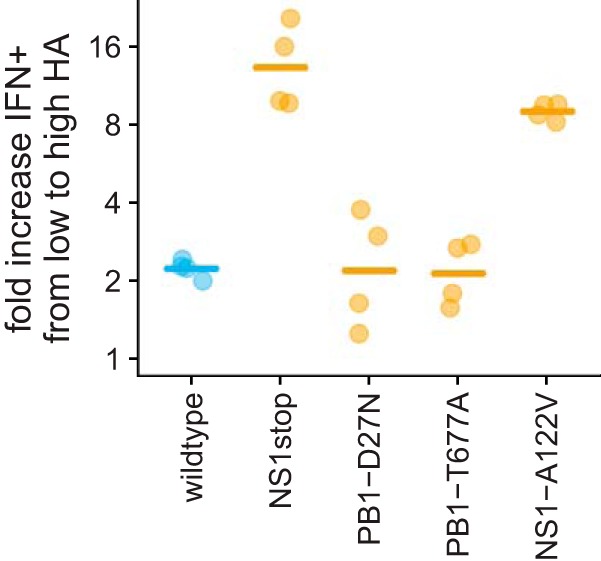
Infected cells that express higher levels of HA protein are much more likely to induce IFN expression only if they are infected by virus with defects in NS1. The *y* axis shows the ratios of the percentage of IFN^+^ cells in the highest HA expression quartile relative to the lowest HA expression quartile. Points indicate replicates, and lines indicate the means. This figure is based on joint analysis of the IFN reporter and HA staining for all infected cells in the flow cytometry data in Fig. S12; see Fig. S14 for more details.

We first focused on one of the strongest trends from the single-cell data: increased viral gene expression was associated with increased IFN induction when the infecting virion failed to express NS, but not otherwise (Fig. 5D). To confirm this observation, we performed a flow cytometry analysis of the reporter cells infected by different immunostimulatory viral mutants to examine the association between expression of a viral gene product (HA protein) and IFN induction. Consistent with the single-cell data, cells that expressed more HA were much more likely to turn IFN^+^ when infected with the NS1stop or NS1-A122V mutants but not when infected with any of the other viral variants (Fig. 7). This fact suggests that when there are high levels of viral transcription, NS1 becomes more important as a buffer against detection of viral products.

**FIG 8:**
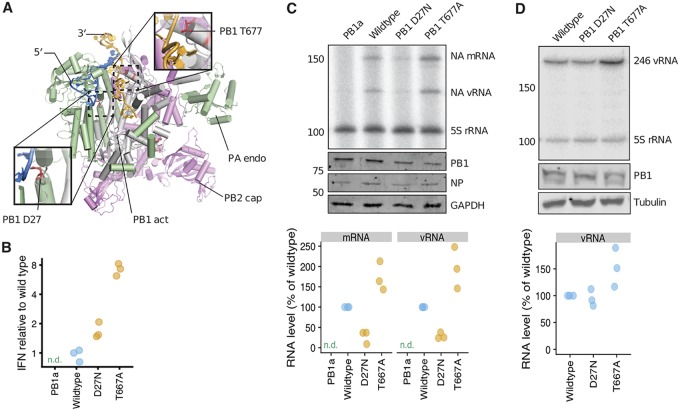
IFN-inducing mutations D27N and T677A in the PB1 protein affect polymerase activity. (A) Model of bat influenza A virus polymerase (PDB 4WSB) (93) superposed with the influenza B virus polymerase (PDB 5MSG) (94). The locations of PB1 D27 and T677 (both red) relative to the 5′ (blue) and 3′ (orange) termini of the RNA template and the PB1 active site (gray; PB1 act) are indicated. The PA endonuclease (green; PA endo) and PB2 cap binding domain (pink; PB2 cap) are also indicated. Part of the fingers subdomain of PB1 is hidden to reveal the template in the entry channel. (B) IFN-β promoter activity measured using a dual luciferase reporter assay in 293T cells transfected with plasmids expressing the indicated PB1 protein, the other polymerase complex proteins (PB2, PA, and NP), and a full-length NA vRNA template. PB1a is a catalytically inactive PB1 active site control. In this panel and the next two panels, points show three biological replicates; “n.d.” indicates not detectable, and orange indicates a variant was significantly different than wild type by a two-sided *t* test. (C) Polymerase activity on full-length vRNA template in 293T cells transfected as in panel B. Steady-state RNA levels were measured by primer extension, denaturing PAGE, and phosphorimaging. PB1a was used as negative control and background correction. The 5S rRNA signal was used as loading control. Other panels show Western blot analysis of PB1, NP, and GAPDH (glyceraldehyde-3-phosphate dehydrogenase) protein expression, and the graph at the bottom shows quantification by phosphorimaging. (D) Polymerase activity on a short 246-nucleotide vRNA template. The top panel shows the steady-state levels of vRNA template as determined by primer extension and denaturing PAGE. The other two panels show the PB1 and tubulin expression levels analyzed by Western blotting, and the graph shows quantification.

We hypothesized that the immunostimulatory mutations to PB1 might cause the viral polymerase to produce aberrant products, in line with recent work showing that mutations to PB2 can lead to the generation of aberrant RNAs that trigger RIG-I (35, 36). To investigate if the PB1 mutations might perturb polymerase activity, we examined their location in a structural model of the polymerase complex (Fig. 8A). The IFN-enhancing PB1 mutation T677A occurs at the tip of a helix that interacts with the 3′ terminus of the RNA template as it enters the channel above the active site, whereas the IFN-enhancing D27N mutation is deeper in the polymerase, close to the binding pocket of the 5′ terminus of the template. Therefore, both mutations could plausibly alter the polymerase’s interactions with the RNA template.

To test if the PB1 mutations affect activity, we transfected 293T cells with plasmids that express wild-type or mutant PB1 protein along with the other proteins in the polymerase complex (PB2, PA, and NP) and full-length viral RNA (vRNA) for the NA segment. Both polymerase mutations increased IFN expression in this assay (Fig. 8B), indicating that they have an immunostimulatory effect in the context of an active viral polymerase even when other viral components are absent. We next directly measured polymerase activity on the full-length vRNA template by extracting total RNA and quantifying replication (vRNA) and transcription (mRNA) products by primer extension. Both immunostimulatory PB1 mutations had activities that were significantly different from those of the wild type, despite being expressed at wild-type protein levels (Fig. 8B). Specifically, the T677A mutant had higher levels of both activities, whereas the D27N mutant had reduced levels of both—although D27N still retained activity far in excess of that of a control active-site mutant (Fig. 8C). We speculated that the mutations might alter polymerase processivity, leading to accumulation of aberrant RNA products that activate the innate immune system (35, 38–42). We therefore repeated the activity assays using a short 246-nucleotide template (35) in place of the full-length NA vRNA (Fig. 8D). On this shorter template, the activity of the D27N mutant was now similar to that of the wild type, while the activity of the T677A mutant remained higher than that of the wild type (although not significantly so in three biological repeats). Therefore, the two immunostimulatory PB1 mutations have distinct effects on the polymerase: D27N reduces processivity thereby favoring shorter RNA products, whereas T677A increases overall activity which could also lead to accumulation of aberrant RNA products.

Overall, the results in this section show that the diverse range of immunostimulatory viral defects identified in single cells act by diverse processes, demonstrating that viral variation influences not only the rate of IFN induction but also the factors that contribute to this induction.

## DISCUSSION

We have determined the full sequences of all viral genes in single influenza virus-infected cells. Methodologically, our major advance is to measure the genotypes of viruses in addition to the abundance of viral components (i.e., transcripts, proteins, or progeny virions) as has been done by prior single-cell studies (12–16, 43, 45–48, 75–77). Our method builds on the observation that fragmentary viral genetic information can be obtained by more standard single-cell transcriptomic techniques (16, 46, 47). To make this information complete, we have coupled single-cell transcriptomics with long-read PacBio sequencing of viral genes, a strategy analogous to that used previously (78) to obtain full-length isoforms of some cellular genes in single cells.

This viral genetic information helps explain cell-to-cell variation in viral gene expression and innate immune induction. Despite the fact that we used a low-passage-number viral stock generated from plasmids, most infected cells did not express unmutated copies of all viral genes. Although our study is certainly not the first to note that influenza virus has a high mutation rate (30–34) and sometimes fails to express genes (12, 15, 43, 61, 62), it is the first to directly observe the full spectrum of these defects across single cells. Visual inspection of Fig. 4 shows how any experiment that does not sequence viral genes in single cells is averaging across a diverse spectrum of viral defects.

We identified four types of defects that we validated to increase IFN induction. Two types of defects—absence of the NS gene and amino acid mutations to the NS1 protein—presumably impair NS1’s well-known ability to antagonize innate immunity (24, 25). Although the general role of NS in innate immune antagonism has long been appreciated, our work represents the first direct demonstration that stochastic absence and mutations to this gene are a major contributor to IFN induction in single cells. A third type of defect, amino acid mutations in PB1, was more surprising since this protein has not been described as a major player in innate immune detection. We characterized two IFN-inducing PB1 mutations and showed that one impaired polymerase processivity whereas the other increased overall activity. We speculate that these alterations increase production of aberrant immunostimulatory RNA products (35). Finally, we found an internal deletion in PB1 that enhances IFN induction, consistent with prior work showing such deletions are immunostimulatory (38–42). In fact, given the extensive prior work on deletions, we were surprised not to identify more of them in our IFN^+^ cells. There may be several reasons: we used pure viral stocks (54) at modest MOI, our experiments preferentially captured cells with higher viral transcriptional load, and most prior studies have used techniques that can detect large deletions but not subtle point mutations. Additionally, the relative importance of different defects likely varies across infection conditions, viral strains, and cell types: it is an open question which defects are most relevant for immune detection during actual human infections.

However, the greatest value of our work is not as a screen for IFN-inducing defects but rather as a relatively unbiased survey of the breadth of viral variation in individual infected cells. This survey shows that no single type of viral defect determines whether a cell induces IFN: even the most immunostimulatory defect (absence of NS) occurs in only approximately one-quarter of IFN^+^ cells. Therefore, innate immune detection of influenza is a multifaceted process that cannot be ascribed a single dominant viral genetic cause.

Our results further show that viral genetic defects do not fully explain the heterogeneity among influenza virus-infected cells. There is substantial breadth in viral transcriptional burden and occasional IFN induction even among cells infected with unmutated virions. Additionally, no viral defect induces IFN deterministically: every type of immunostimulatory defect that we characterized is also observed in IFN^−^ cells in our single-cell data set. Therefore, stochasticity or preexisting cellular states also play a major role in affecting innate immune induction, a finding that concords with the fact that IFN induction is heterogeneous even among cells treated with synthetic innate immune ligands (20–23), as well as for other viruses (48).

Perhaps the most intriguing question is how the heterogeneity that we have described ultimately affects the macroscopic outcome of infection. Natural human influenza virus infections are established by just a few virions (17–19) that then undergo exponential growth, and early IFN responses are amplified by paracrine signaling (1, 2). It is therefore plausible that early heterogeneity in innate immune induction could affect the entire course of infection. Extending our approaches to more complex systems could shed further light on how viral genetic variation and stochasticity interact to shape the race between virus and immune system.

## MATERIALS AND METHODS

### IFN reporter cell lines.

We created IFN reporter variants of the A549 human lung epithelial cell line (Fig. 1A). The parental A549 cell line used to create these reporters was obtained from ATCC (CCL-185) and was tested as negative for mycoplasma contamination by the Fred Hutch Genomics Core and authenticated using the ATCC STR profiling service. The cells were maintained in D10 medium (Dulbecco’s modified Eagle medium [DMEM] supplemented with 10% heat-inactivated fetal bovine serum, 2 mM l-glutamine, 100 U of penicillin/ml, and 100 μg of streptomycin/ml) at 37°C and 5% carbon dioxide.

To create the type I interferon reporters, a 1-kb promoter region upstream of the human *IFNB1* gene was cloned into the pHAGE2 lentiviral vector (79), with a NotI site immediately downstream of the promoter serving as an artificial Kozak sequence. Downstream of this NotI site, each of the following reporter constructs was cloned: mCherry, mNeonGreen, and low-affinity nerve growth factor lacking the C-terminal signaling domain (LNGFRΔC) (50, 51) linked to mNeonGreen by a P2A linker (80). The sequence of the last of these constructs is provided in File S1 in the supplemental material.

To create the type III interferon reporters, a 1.2-kb region upstream of the human interleukin 29 (IL-29) gene (*IFNL1*) was cloned into the pHAGE2 vector, with the native Kozak sequence retained at the 3′ end. Downstream of this promoter we cloned LNGFRΔC linked to ZsGreen via a P2A linker. The sequence of this construct is provided in File S1.

We used these constructs to generate lentiviral vectors and transduce A549 cells in the presence of 5 μg Polybrene. We then sorted single transduced cells and expanded them. A portion of the expanded cells were tested for reporter activity by transfecting poly(I·C) (a potent agonist of the RIG-I pathway), and we retained clones with strong activation. Importantly, the cells that we retained for further use were not the same portion that were tested by poly(I·C) treatment but rather a separate split of the same population—this avoids any selection on the cells from transient activation of IFN. For the dual-type I/type III reporter used in Fig. S1B, a single-cell clone of the type III reporter cell line was transduced with the type I reporter bearing the mCherry fluorescent marker and then isolated and propagated as a single cell clone for the other cell lines. All reporter lines tested negative for mycoplasma contamination by the Fred Hutch Genomics Core.

Figure S1A shows validation of the reporter cell lines using infection with saturating amounts of the Cantell strain of Sendai virus (obtained from Charles River Laboratories). For detection of the cell surface-bound LNGFRΔC, cells were stained with phycoerythrin (PE)-conjugated anti-LNGFR (CD271) antibody from Miltenyi Biotec.

### Viruses for single-cell experiments.

We performed the single-cell experiments using the A/WSN/1933 (H1N1) strain of influenza virus. We used both the wild-type virus and a variant of the virus in which synonymous mutations were added within a few 100 nucleotides of each termini of each gene segment. We have used a similar synonymous viral barcoding strategy in our prior single-cell work (12) as it allows us to detect approximately one-half of coinfected cells based on the expression of both viral barcode variants. In the present work, we extended this approach by placing synonymous barcodes near both termini of the gene segments in order to quantify strand exchange during PacBio sequencing (Fig. S10). The sequences of all gene segments from the wild-type and synonymously barcoded viral strains are in File S2. These genes were cloned into the pHW2000 (53) reverse genetics plasmid.

Both viral strains were generated by reverse genetics using the pHW18* series of bidirectional plasmids (53). We controlled the durations and MOI during viral passaging, since these factors can greatly affect the accumulation of defective viral particles (54). The viruses were generated by reverse genetics in cocultures of 293T and MDCK-SIAT1 cells in influenza growth medium (Opti-MEM supplemented with 0.01% heat-inactivated fetal bovine serum [FBS], 0.3% bovine serum albumin [BSA], 100 U of penicillin/ml, 100 μg of streptomycin/ml, and 100 μg of calcium chloride/ml) and then propagated in MDCK-SIAT1 cells in influenza growth medium using the same basic procedures detailed in reference 12. Specifically, after generation by reverse genetics, the wild-type variant was expanded at an MOI of 0.001 for 72 h twice in MDCK-SIAT1 cells, and the synonymously barcoded variant was expanded once at an MOI of 0.01 for 60 h. The MOIs for this passaging are based on titers determined using 50% tissue culture infective dose (TCID_50_) assays via the formula of Reed and Muench (81) as implemented at https://github.com/jbloomlab/reedmuenchcalculator. After being passaged independently, the two viral stocks were combined at equivalent numbers of infectious units to make the stock used in the single-cell experiments.

### Flow cytometry analyses for HA expression.

For the single-cell experiments (which only examined the transcriptional results of a single cycle of infection), we were most interested in the titer of viral particles that were transcriptionally active for a single round of infection of A549 cells. We estimated titers of transcriptionally active virions by staining for HA expression in virus-infected A549 cells. Specifically, we infected A549 cells (or one of the A549 reporter cell line variants as described above) in influenza growth medium, and at 13 to 14 h postinfection, we trypsinized cells, resuspended them in phosphate-buffered saline (PBS) supplemented with 2% heat-inactivated fetal bovine serum (FBS), and stained them with 10 μg/ml of H17-L19, a mouse monoclonal antibody previously shown to bind to the HA from the A/WSN/1933 strain of virus (82). After washing in PBS supplemented with 2% FBS, the cells were stained with a goat anti-mouse IgG antibody conjugated to allophycocyanin (APC), washed, fixed in 1% formaldehyde in PBS, washed again, and then analyzed by flow cytometry to determine the fraction expressing detectable HA protein.

### Single-cell transcriptomics of IFN-enriched infected cells using 10x Chromium.

The single-cell transcriptomics and virus sequencing were performed using the A549 cells with the *IFNB1* LNGFRΔC-P2A-mNeonGreen reporter. A schematic of the experiment is shown in Fig. 2.

The wild-type and synonymously barcoded viruses were mixed with the goal of adding equal numbers of transcriptionally active HA-expressing virions of each virus strain. The cells were then infected with this mixture at a dose designed to infect approximately one-half of the cells (Fig. 3C suggests that the actual rate of detectable infection was slightly lower). Infections were allowed to proceed for 12 h. The cells were then trypsinized, the trypsin was quenched with D10 medium, and cells were resuspended in degassed PBS supplemented with 0.5% bovine serum albumin and 5 mM EDTA. To enrich IFN^+^ cells, the cells were then incubated with anti-LNGFR MACSelect microbeads (Miltenyi Biotec) and twice passed over an MS magnetic column (Miltenyi Biotec), retaining the bound (and presumably IFN-enriched) population each time.

This MACS sorting is expected to give approximately the enrichment for IFN^+^ cells shown in Fig. S3. The original unsorted population was then added back in to ∼10% of the final cell fraction in order to ensure the presence of interferon-negative cells. At this point, uninfected canine (MDCK-SIAT1) cells were also added to ∼5% of the final cell fraction to enable quantification of the cell multiplet rate (Fig. 3A) and background viral mRNA in uninfected cells (Fig. 3C). We began this entire process of cell collection and enrichment at 12 h postinfection, but the process (which was performed at room temperature) took approximately 1 h; thus, we consider the cells to have been analyzed at 13 h postinfection. The final cell suspension was counted using a disposable hemocytometer and loaded on the 10x Genomics Chromium instrument (55), targeting capture of ∼1,500 cells.

This sample was then processed to create libraries for Illumina 3′-end sequencing according to the 10x Genomics protocol using the Chromium Single Cell 3′ Library and Gel Bead kit v2 with one important modification: rather than process all full-length cDNAs through enzymatic fragmentation, several nanograms were retained for targeted full-length viral cDNA sequencing as described below. The single-cell transcriptomics library was sequenced on an Illumina HiSeq 2500, and the data were analyzed as described below.

### Enrichment and preparation of viral cDNA for PacBio sequencing.

We amplified virus-derived molecules from cDNA retained from the 10x Genomics protocol for PacBio sequencing of the full-length cDNA. These cDNAs have at their 3′ ends the cell barcode and UMI plus the adaptor sequence that is added during the 10x protocol (see Fig. 2 for simple schematic, and File S7 for more details). We only wanted to PacBio sequence cDNA molecules derived from virus. We therefore needed to enrich for the viral molecules while retaining the 10x adaptor/UMI/cell barcode at the 3′ end.

We first performed a multiplex PCR on 1 ng of the full-length 10x cDNA using a 3′ primer complementary to the common 10x adaptor and a multiplex mix of eight 5′ primers, one specific for the mRNAs from each of the eight viral gene segments (File S3). A major concern during these PCRs is strand exchange (see Fig. S10), which would scramble the cell barcodes and mutations on viral cDNAs. To reduce strand exchange and obtain more even PCR amplification across segments, we performed emulsion PCRs using the Micellula DNA Emulsion kit (Roboklon), which physically separates disparate template molecules, preventing strand exchange and allowing each molecule to be amplified to exhaustion of its droplet’s reagents (83). We performed the PCRs using Kapa HiFi Hotstart ReadyMix, supplementing the reactions with additional BSA to a final concentration of 0.1 mg/ml and using a volume of 100 μl. Both the common 3′ primer and the multiplex mix of eight 5′ primers were added to a final concentration of 0.5 μM. We performed 30 cycles of PCR, using an extension time of 2 min 15 s at 67°C and a melting temperature of 95°C. This melting temperature is lower than the standard 98°C melting step suggested by the manufacturer for Kapa HiFi because we wanted to avoid collapse of emulsion integrity at high temperature.

The product of this multiplex PCR was subjected to eight additional individual emulsion PCRs, each using only a single segment-specific 5′ primer as well as the common 3′ primer, using 1 ng of material in each reaction. The material from these eight segment-specific PCRs was then pooled with the goal of obtaining an equimolar ratio of segments and sequenced on one SMRT Cell in a PacBio RS II and one SMRT Cell of a PacBio Sequel. Detailed results from the analysis of these first two sequencing runs are shown in File S7. These results showed that although the PCRs substantially enriched for influenza virus molecules, the relative coverage of the different viral genes was still uneven, with the longer genes undersampled.

To improve coverage of the polymerase genes, we produced two new sequencing pools: one consisting of the five shortest viral segments (HA, NP, NA, M, and NS) from the aforementioned segment-specific emulsion PCRs and the other consisting of the three longer polymerase segments (PB2, PB1, and PA). The former was sequenced on one cell of a single SMRT Cell of a PacBio Sequel and the latter on two additional SMRT Cells of a PacBio Sequel. As is shown File S7, the coverage remained relatively low for the polymerase genes, and most of the reads we did obtain were dominated by shorter internally deleted variants of the polymerase genes (54) which are preferentially amplified during PCR.

To obtain more reads for longer full-length polymerase variants, we therefore subjected 10 ng of our amplified material for each polymerase segment to a bead selection using SPRIselect beads at a volume ratio of 0.4. This selection removes most low-molecular-weight DNA species, including internally deleted defective segments. Material from this selection was amplified using 16 (PB1) or 14 (PB2 and PA) cycles of a nonemulsion PCR using the standard conditions recommended by the Kapa HiFi Hotstart ReadyMix (extension at 67°C for 2 min 15 s and melting at 98°C). The use of relatively few PCR cycles was designed to prevent the occurrence of the artifacts (including strand exchange) that occur in nonemulsion PCRs. We pooled the products of these reactions from this size selection and sequenced on a SMRT Cell of a PacBio Sequel. As is shown in File S7, this sequencing yielded more full-length polymerase variants, but they were still undersampled compared to other viral genes. To further improve recovery of full-length PB1, PB2, and PA, we used an approach that allowed us to perform a specific PCR for full-length polymerase variants. We circularized the template molecules and then used two segment-specific primers that annealed in apposition near the center of each polymerase gene to linearize these circular molecules. Only molecules that contain the middle of the polymerase genes (which are typically full length) are linearized by this process. In the downstream computational analysis, we can then determine the full sequence of the gene as well as the cell barcode of the initial molecule from which the linearized molecule is derived. Specifically, we first used 2.5 ng of our already-amplified segment-specific material in a 10-cycle PCR to append circularization adapters (see File S3 for sequences) and cleaned the resultant mixture using SPRIselect beads at a volume ratio of 0.4. We then used 10 ng of this amplified material in a 20-μl NEBuilder reaction mixture using an extended reaction time of 50 min in order to circularize the molecules. We next incubated these reactions for 1 h at 37°C with exonuclease V and additional ATP to a final increase in concentration of 1 mM to digest all noncircularized molecules. The circularized and digested material was then cleaned using SPRIselect beads at a volume ratio of 0.4. This material was then used as the template for three nonemulsion PCRs specific to PB2, PB1, or PA, using two segment-specific primers that align to the central portion of each gene but in apposition to each another (see File S3 for sequences). These linearization reactions used 20 (PB2) or 26 (PB1 and PA) PCR cycles, and the resulting products were cleaned using SPRIselect beads at a volume ratio of 1.0. This material was pooled to produce an equimolar mixture of full-length PB1, PA, and PB2 and sequenced in an additional SMRT Cell of PacBio Sequel. As is shown in File S7, this process yielded many full-length polymerase variants.

The computational analyses of the full-length viral gene sequences described below combined the data from all these reactions. The number of sequences obtained for each gene after pooling the data from all reactions is shown in Fig. S9, which also indicates that the net rate of strand exchange is very low (see Fig. S10 for an illustration of how this is determined). A detailed breakdown of the coverage of each gene and PacBio run is in File S7. Importantly, the PCR biases mean that the coverage of molecules by the PacBio sequencing was not proportional to their abundance in the starting mRNA. However, as described in the computational analysis section below, the final analyses used the cell barcodes and UMIs in conjunction with the standard 10x Illumina sequencing to ensure that none of the conclusions were affected by the disproportionate amplification of some molecules during the PacBio library preparation (for instance, duplicate UMIs were removed from the PacBio data, and all conclusions about gene abundance or absence were based on the Illumina data).

### qPCR for viral genes and IFN.

We performed quantitative PCR (qPCR) on reverse-transcribed mRNA for influenza virus HA (to quantify viral transcription), IFNB1 (to quantify IFN induction), and L32 (a cellular housekeeping gene for normalization). For the qPCR, we used the SYBR green PCR Master Mix (Thermo Fisher) according to the manufacturer’s protocol using oligo(dT) primers. The qPCR primers were as follows: HA primer 1, 5′-GGCCCAACCACACATTCAAC-3′; HA primer 2, 5′-GCTCATCACTGCTAGACGGG-3′; IFNB1 primer 1, 5′-AAACTCATGAGCAGTCTGCA-3′; IFNB1 primer 2, 5′-AGGAGATCTTCAGTTTCGGAGG-3′; L32 primer 1, 5′-AGCTCCCAAAAATAGACGCAC-3′; L32 primer 2, 5′-TTCATAGCAGTAGGCACAAAGG-3′. For the qPCR in Fig. S13, A549 cells were seeded at a density of 10^4^ cells/well in a 96-well plate in D10 medium 24 h prior to infection, with four independent wells seeded per experimental treatment. Immediately prior to infection, D10 medium was removed and replaced with influenza growth medium and cells were infected with the various influenza strains at an MOI of 0.4 based on TCID_50_ in MDCK-SIAT1 cells. For the cells with cycloheximide added to block protein expression (and hence secondary transcription), cycloheximide was added to a final concentration of 50 μg/ml (a concentration sufficient to block secondary transcription [84]) at the time of infection. After 8 h, mRNA was harvested using the CellAmp Direct RNA Prep kit for reverse transcription-PCR (RT-PCR) and reverse transcribed using an oligo(dT) primer, and qPCR was performed as described above.

### Viruses and experiments for validation experiments.

As shown in Fig. 6, we tested the IFN inducing capacity of a variety of viral mutants identified in the single-cell experiments. For point mutant viruses, we created variants for all amino acid substitutions found in PB1 and NS among IFN^+^ cells that did not also lack NS. One of these mutants (amino acid substitution S704P in PB1) did not reach sufficient titers in a single attempt to generate it by reverse genetics, and so it was dropped from the experiment (note that we did not attempt replicates of the reverse genetics for this mutant, and so we are not confident in drawing strong conclusions about its actual attenuation). This left six point mutant viruses: four with point mutations in PB1, and two with point mutations in NS. We also created a mutant virus that contained the internal deletion in PB1 found in an IFN^+^ cell. In addition, we created a virus with an inactivated NS1 to mimic the infections that failed to express NS (we were unable to use complementing cells to generate a viral stock that completely lacked the NS segment). This NS1stop virus contained six nucleotide changes resulting in the addition of five in-frame stop codons in NS1 starting 10 nucleotides downstream of the 5′ splice donor site, thereby disrupting NS1 while leaving NS2 (NEP) intact. All of these mutants were cloned into the pHW2000 bidirectional reverse genetics plasmid (53) in order to enable generation of viruses encoding the mutant genes. File S6 provides the full sequences for all of these plasmids.

We generated the wild-type and point mutant viruses for the validation experiments shown in Fig. 6A by reverse genetics using the pHW18* series of WSN reverse genetics plasmids (53) but substituting the appropriate mutant plasmid listed in File S6 for the wild-type plasmid for that gene. To generate the viruses from these plasmids, we transfected cocultures of 293T and MDCK-SIAT1 cells seeded at a ratio of 8:1 with an equimolar mix of all eight plasmids. At 24 h posttransfection, we changed medium from D10 to influenza growth medium. At 50 h posttransfection (for the replicate 1 viruses in Fig. S12A) or 72 h (for the replicate 2 viruses in Fig. S12), we harvested the virus-containing supernatant, clarified this supernatant by centrifugation at 300 × *g* for 4 min, and stored aliquots of the clarified viral supernatant at −80°C. We then thawed aliquots and determined the titer by TCID_50_ on MDCK-SIAT1 cells. For the infections shown in Fig. S12A, we wanted to use equivalent particle counts, and so we normalized all viruses to an equivalent hemagglutination titer on turkey red blood cells (85). Briefly, a solution of 10% (vol/vol) red blood cells (LAMPIRE Biological Laboratories, Fisher Scientific catalogue number 50412942) was washed in PBS and diluted to a final concentration of 0.5% (vol/vol). Two-fold serial dilutions of virus were added to equal volumes of diluted red blood cells, and the titers were measured as the highest dilution of viral stock at which complete hemagglutination of red blood cells was observed. We then performed infections of the A549 reporter cell line at an equivalent hemagglutination titer and analyzed the data as described for Fig. S12A.

To generate the NS1stop mutant virus and the wild-type and PB1del385to2163 mutant viruses shown in Fig. S12B, we used slightly different procedures. The wild-type virus was generated by reverse genetics as described above for the point mutant viruses, harvested at 48 h posttransfection, and then passaged on MDCK-SIAT1 cells for 36 h at an MOI of 0.05—conditions that we previously validated to lead to relatively little accumulation of defective particles (12). The NS1stop virus was similarly generated but was passaged for 48 rather than 36 h, since it had slower growth kinetics and so needed a longer period of time to reach high titers. The viruses with deletions in the PB1 segment could not be generated in normal 293T and MDCK-SIAT1 cells, since they required the exogenous expression of the PB1 protein. Therefore, these viruses were generated in previously described 293T and MDCK-SIAT1 cells that had been engineered to constitutively express PB1 (86). These viruses were harvested from transfections at 72 h and passaged twice in the MDCK-SIAT1 cells constitutively expressing PB1 at an MOI of 0.001 for 72 h and 0.01 for 48 h. This passaging was necessary as viral titers from transfections were too low to generate sufficient virus from a single passage. The titers of wild-type and NS1stop viruses were determined by TCID_50_ on MDCK-SIAT1 cells, and the titers of PB1 deletion viruses were determined on the MDCK-SIAT1 cells constitutively expressing PB1. The infections shown in Fig. S12B were performed at equivalent TCID_50_s as described in the legend to that figure. That these equivalent TCID_50_s were also roughly equivalent in terms of particles capable of undergoing primary transcription is shown in Fig. S13.

### Computational analysis of single-cell transcriptomic and viral sequence data.

A computational pipeline that performs all steps in the data analysis is available at https://github.com/jbloomlab/IFNsorted_flu_single_cell. This pipeline is orchestrated by Snakemake (87) and begins with the raw sequencing data and ends by generating the figures shown in this paper. The sequencing data and annotated cell-gene matrix are available on the GEO repository under accession GSE120839 (https://www.ncbi.nlm.nih.gov/geo/query/acc.cgi?acc=GSE120839).

Briefly, the raw deep-sequencing data from the Illumina 3′-end sequencing were processed using the 10x Genomics software package cellranger (version 2.2.0). We built a multispecies alignment reference consisting of a concatenation of the human and influenza virus transcriptomes (the first “species”) and the canine transcriptome (the second “species”). The human transcriptome was generated by filtering genome assembly GRCh38 for protein-coding genes defined in GTF file GRCh38.87. The influenza virus transcriptome consisted of the mRNAs for the wild-type A/WSN/1933 virus strain in File S2 (the cellranger alignment is sufficiently permissive that it aligns sequences from both the wild type and synonymously barcoded viral variants to this transcriptome). The canine transcriptome was generated by filtering genome assembly CanFam3.1 for protein-coding genes defined in GTF file CanFam3.1.87. The cellranger software was used to align the Illumina 3′-end sequencing reads to this multispecies transcriptome, call “human+influenza” and canine cells (Fig. 3A), and generate a matrix giving the expression of each gene in each single cell. We used a custom Python script to determine the number of influenza virus reads that could be assigned to the wild-type or synonymously barcoded virus and added this information to the annotated cell-gene matrix.

The PacBio sequences of the full-length viral genes were analyzed as follows. First, we used version 3.1.0 of PacBio’s ccs program (https://github.com/PacificBiosciences/unanimity) to build circular consensus sequences (CCSs) from the subread files, requiring at least 3 passes and a minimum accuracy of 0.999. We further processed these CCSs using custom Python code and the minimap2 (88) long-read aligner (version 2.11-r797). The Python code has been implemented in the API of dms tools2 (https://jbloomlab.github.io/dms_tools2/) software package (version 2.3.0) (89). A Jupyter notebook that performs these analyses is at https://github.com/jbloomlab/IFNsorted_flu_single_cell/blob/master/pacbio_analysis.ipynb, and is also provided as File S7. We refer the reader to this notebook for a detailed description and extensive plots showing the results at each step. Here is a brief summary: we filtered for CCSs that had the expected 5′ termini (from the influenza virus-specific primers) and 3′ termini (corresponding to the 10x adaptor), and for which we could identify the cell barcode, UMI, and poly(A) tail. We aligned the cDNAs flanked by these termini to the influenza virus transcriptome and performed a variety of quality control steps. At this point, we examined whether cDNAs had the synonymous viral barcodes at both ends or neither end as expected in the absence of strand exchange (Fig. S10) and reassuringly found that strand exchange was rare (Fig. S9). The small numbers of CCSs with identifiable strand exchange were filtered from further analysis. We then further filtered for CCSs that contained valid cell barcodes as identified by the cellranger pipeline and kept just one CCS per UMI (preferentially retaining high-quality CCSs that aligned to full-length cDNAs). We then removed from the CCSs the barcoding synonymous mutations that we had engineered into one of the two viral variants. Finally, we used the CCSs to call the sequence of the viral gene in each cell, calling mutations separately for each viral barcode variant. We called mutations (insertions, deletions, and substitutions) in the viral gene sequences as follows.

- Mutations with accuracies less than 0.999 (which constitute <0.5% of all mutations) were ignored.
- If all CCSs for a particular viral barcode variant of a gene in a cell were wild type, it was called wild type.
- If any CCSs for a particular viral barcode variant of gene in a cell had a mutation, then at least two CCSs were required to call the sequence.
- If at least two and >30% of the CCSs had a specific mutation, then that mutation was called present and its frequency noted among the CCSs. The exception was single-nucleotide indels in homopolymers, for which we required three CCSs to call a mutation (the reason is that the main mode of PacBio sequencing errors is short indels in homopolymers).

The plots in File S7 indicate that these are reasonable mutation-calling criteria. We could call the sequences of all expressed viral genes in approximately one-half of the infected cells (Fig. S11). The mutations called using this pipeline are shown in Fig. 4, and File S4 gives the number of CCSs supporting each mutation call. The called sequences of the viral genes were added to the annotated cell-gene matrix.

Finally, we process the annotated cell-gene matrix in R to generate the plots shown in this paper. This analysis utilized a variety of R and Bioconductor (90) packages, including Monocle (91, 92) and ggplot2. A Jupyter notebook that performs these analyses is at https://github.com/jbloomlab/IFNsorted_flu_single_cell/blob/master/monocle_analysis.ipynb, and is also provided as File S8. We refer the reader to this notebook for a detailed description and a variety of additional plots not included in the paper. Briefly, we first filtered cells that were extreme outliers in the amount of mRNA as shown in Fig. 3B. We used the uninfected canine cells to estimate the percentage of total mRNA in a cell that would come from influenza virus purely due to background (e.g., from cell lysis) in the absence of infection, and called as infected the human cells for which significantly more than this amount of mRNA was derived from influenza under a Poisson model (Fig. 3C). We next used a Poisson model parameterized by the amount of expected background mRNA for each influenza gene to call the presence or absence of each influenza gene in each infected cell (Fig. 3D and Fig. S4). To identify cells that were coinfected with both viral barcodes (Fig. 3F), we used a binomial test to identify cells for which we could reject the null hypothesis that at least 95% of viral mRNA was derived from the more common viral barcode. We called IFN^+^ and ISG^+^ cells using the heuristic thresholds shown in Fig. 3G and Fig. S6, respectively. We counted IFN mRNAs as any alpha, beta, or gamma interferon (IFN-α, IFN-β, or IFN-λ, respectively) transcripts. We counted ISG mRNAs as any of CCL5, IFIT1, ISG15, or Mx1. The plot in Fig. 4 summarizes all of the genotypic information and was created in substantial part using gggenes (https://github.com/wilkox/gggenes). The raw data are in Files S4 and S5.

### Structural analysis of PB1 mutants.

To locate the PB1 mutations in the influenza A virus RNA polymerase structure relative to the template and active site shown in Fig. 8A, we superposed the bat influenza A virus RNA polymerase structure (PDB 4WSB) (93), which shows the 3′ terminus of the template on the surface of the RNA polymerase, with the influenza B virus transcription initiation complex (PDB 5MSG) (94), which shows the 3′ terminus of the template in the template entry channel that leads toward the active site. The structural alignment was performed in PyMOL 1.8.7 using motifs A and C.

### Experimental analysis of PB1 mutants.

For the experimental analysis of the PB1 mutants shown in Fig. 8, we used plasmids pcDNA-PB1, pcDNA-PA, pcDNA-PB2, and pcDNA-NP, which encode the WSN proteins that compose the polymerase complex (95), pPolI-NA, which encodes the viral RNA for the WSN NA (95), and pcDNA-PB1a, which encodes an inactive version of the WSN PB1 polymerase protein (96). To construct plasmids expressing mutant PB1 proteins D27N and T677A, the plasmid pcDNA-PB1 was subjected to site-directed mutagenesis. PB1 expression was analyzed by Western blotting using antibody GTX125923 (GeneTex).

To analyze the activity of the PB1 mutants in cell culture, the plasmids expressing the WSN PA, PB2, NP, and PB1 proteins were transfected into 293T cells together with the plasmid expressing the wild-type NA vRNA or a 246-nucleotide (nt)-long segment NP-based template (35). Twenty-four hours posttransfection, the RNA was extracted using Trizol (Invitrogen), and the steady-state RNA levels were assessed using reverse transcription with ^32^P-labeled oligonucleotides against the viral RNA species and ribosomal 5S RNA as described previously (35, 97). ^32^P-derived signals were imaged using phosphorimaging on a Typhoon scanner and analyzed using Prism (GraphPad). In all experiments, the apparent RNA levels were background corrected using the PB1a mutant and normalized to the 5S rRNA loading control.

To measure the induction of the IFN-β promoter during these RNP reconstitution assays, they were carried out in the presence of a plasmid expressing *Renilla* luciferase from a cytomegalovirus (CMV) promoter and a plasmid expressing Firefly luciferase from the IFN-β promoter (35). Twenty-four hours posttransfection, cells were harvested, lysed, and analyzed using a DualGlo luciferase kit (Promega) according to the manufacturer’s instructions. Samples were analyzed using GloMax (Promega).

### Data availability.

Raw data are available on the GEO repository under accession number GSE120839. Computer code is available on GitHub (https://github.com/jbloomlab/IFNsorted_flu_single_cell).

## Supplementary Material

Supplemental file 1
