# Supplementary material for: Single-Cell Virus Sequencing of Influenza Infections That Trigger Innate Immunity
Source: J Virol. 2019 Jun 28;93(14):e00500-19. doi: 10.1128/JVI.00500-19 (PMC6600203; doi:10.1128/JVI.00500-19)
Supplement: Supplemental file 1 [file JVI.00500-19-s0001.pdf]

SUPPLEMENTAL MATERIAL

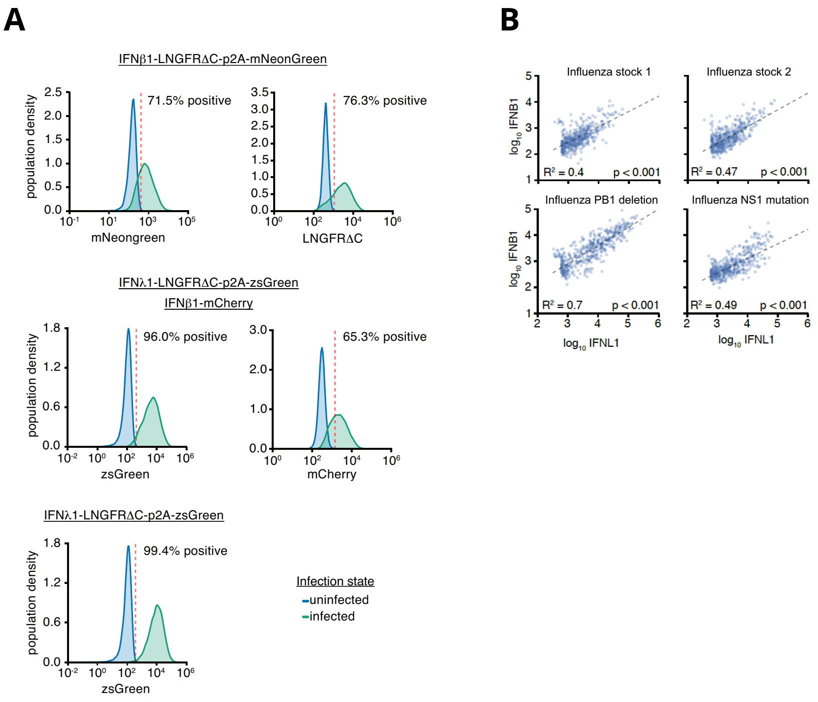

FIG S1 Validation of reporter cell lines (Fig. 1A) to identify IFN+ cells. (A) To validate the IFN reporter cell lines, they were infected at high MOI with the Cantell strain of Sendai virus, which strongly activates IFN expression. The name of each of reporter cell line is indicated at the top of each row of plots. At 13 hours post-infection, activation of the IFN reporter was then monitored by flow cytometry using the marker indicated at the bottom of each plot (either a fluorescent protein or antibody staining for the cell-surface LNGFRΔC using a PE-conjugated anti-LNGFR antibody from Miltenyi Biotec). Sendai infection efficiently activated the IFN reporter in all cases, with the strongest signal from the IFN-λ reporter driving ZsGreen. (B) The type I and type III IFN reporters are highly correlated in their activation. An A549 cell line was generated by transduction with both the IFN-β and IFN-λ reporters driving expression of mCherry and ZsGreen, respectively. The cells were then infected with two different stocks of "wild-type" WSN influenza, or stocks with a deletion in PB1 or stop codons in NS1 (described later in the paper). After 13 hours, cells were analyzed by flow cytometry. Cells positive for either fluorescent reporter were further analyzed. As shown in the FACS plots, expression of the *IFNB1* and *IFNL1* reporters is highly correlated in all cases.

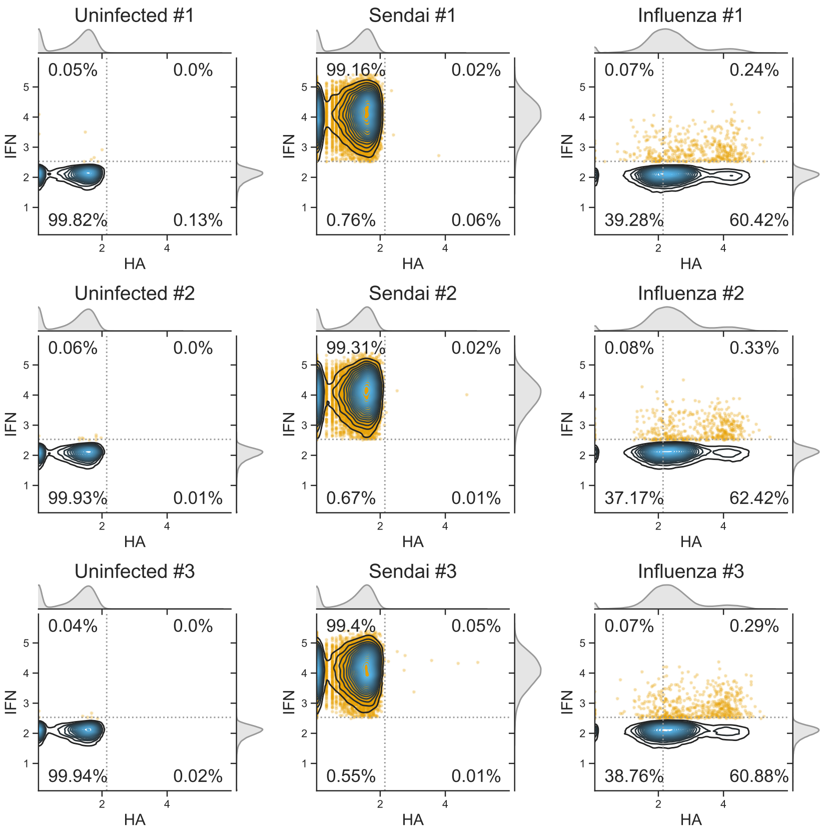

FIG S2 Flow cytometry data for Fig. 1B. The A549 cells with the *IFNL1* reporter driving LNGFRΔC-ZsGreen were not infected, infected with saturating amounts of the Cantell strain of Sendai virus, or infected the same stock of influenza virus used in the single-cell experiment at a target MOI of 0.3. After 13 hours, the cells were stained for expression of HA protein and analyzed by FACS for HA and expression of the ZsGreen driven by the *IFNL1* reporter. Each condition was done in triplicate. The contour plots show the density of all cells, and all IFN+ cells are also indicated by orange dots. Cells were classified as HA+ or IFN+ based on gates set to put 0.05% of the uninfected cells in these populations. For the influenza-infected cells, the percentage IFN+ was calculated only among the HA+ cells (since these are the ones that are infected). For the uninfected and Sendai-virus infected, the percentage IFN+ was calculated among all cells, since these cells do not express HA.

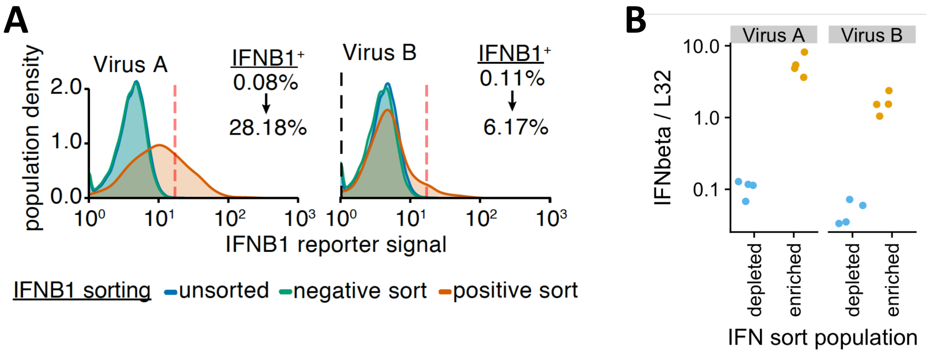

FIG S3 Example MACS enrichments of IFN+ influenza-infected cells. A549 cells with the *IFNB1* LNGFRΔC-mNeonGreen reporter were infected with wild-type WSN influenza (two different viral stocks) at a target MOI of 0.1 TCID50 per cell. After infection had proceeded for 12 hours, the cells were twice magnetically sorted for LNGFRΔC expression over magnetic columns as detailed in the methods for the single-cell sequencing experiment. (A) After sorting, the populations were analyzed by flow cytometry for IFN expression using the mNeonGreen fluorescent protein. The plots show the distribution of fluorescence in the original population, the flow-through from the first column, and the MACS-sorted positive population after two columns. As indicated by the percentages shown for the original and MACS-sorted population, this process led to substantial enrichment in IFN+ cells. We expect that the IFN sorting for the actual single-cell sequencing led to similar enrichment, although we could not directly quantify this as the sorted cells in that case were immediately used for the sequencing and so could not be analyzed by flow cytometry. (B) Analysis of expression of IFNB1 (relative to the housekeeping gene L32) by qPCR in the positive (IFN enriched) and negative (IFN depleted) populations from panel (A). The qPCR validates a roughly 50- to 100-fold enrichment in total IFNB1 expression. The qPCR was performed in quadruplicate (hence the four points for each sample).

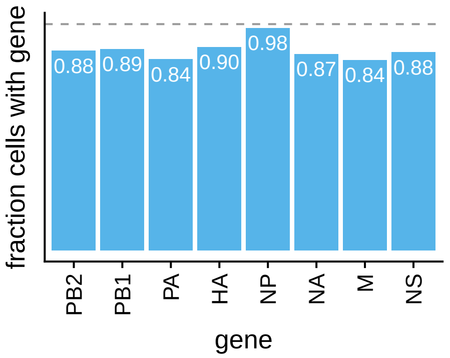

FIG S4 The fraction of infected cells that are called as expressing each viral gene. The gray dashed line is at one (the fraction that would be observed if all viral genes are expressed in all infected cells). Each viral gene is detected in ~80-90% of the infected cells. The exception is NP, which is detected in virtually all infected cells. The much higher frequency of detecting NP could reflect a biological phenomenon, but we suspect it is more likely that cells lacking NP tend to have much lower viral gene expression overall and so are not reliably called as being infected in our experiments because the number of viral mRNAs is below the detection limit.

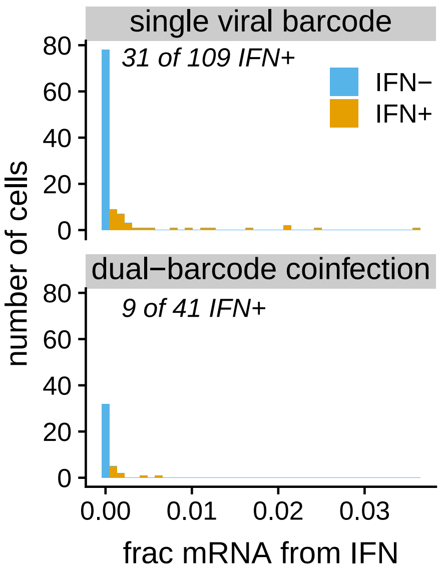

FIG S5 There is no association between viral co-infection and expression of IFN in our experiments using low MOI infections with a relatively "pure" viral stock. Histograms show the fraction of all cellular mRNA derived from IFN among cells expressing viral mRNA from just a single viral barcode variant, or cells expressing viral mRNA from both the wildtype and synonymously barcoded viral variants. The dual-barcode cells represent known co-infections, whereas the single-barcode cells represent a mix of singly infected cells and co-infections with the same viral barcode. There is no significant difference in the frequency of IFN induction among the two classes of cells ($P = 0.53$, Fisher's exact test).

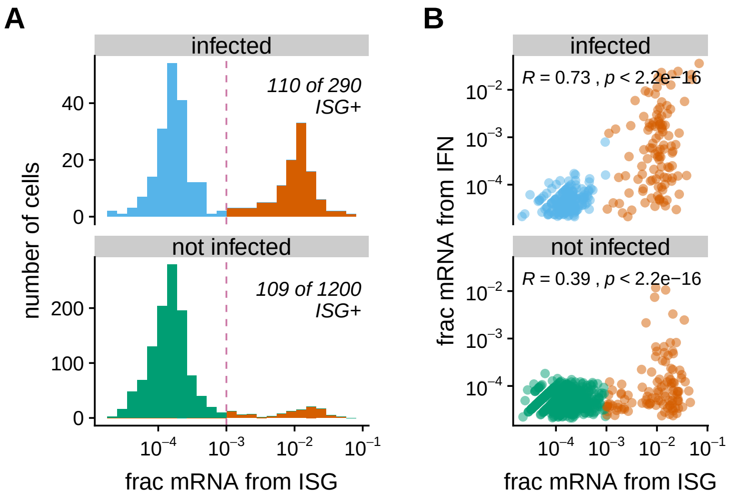

FIG S6 Expression of ISGs in single infected and uninfected cells. For each cell, we quantified ISG expression as the total fraction of cellular mRNAs derived from four prototypical ISGs (IFIT1, ISG15, CCL5, and Mx1). (A) The histograms show the distribution of ISG expression taken across infected (top) and uninfected (bottom) cells. We heuristically classify as ISG+ cells with $> 10^{-3}$ of their cellular mRNA from ISGs, and color these cells red. Comparison to Fig. 3G shows that substantially more cells are ISG+ than IFN+, both among infected and uninfected cells. This is probably because paracrine signaling can induce ISG expression in cells that are not themselves expressing IFN. (B) Correlation between the fraction of cellular mRNA derived from IFN and ISGs. Each point represents one cell, and the Pearson correlation coefficient is shown. IFN and ISG expression are more correlated for infected than uninfected cells, probably because in the latter the ISG expression is more often due to paracrine signaling that does not induce expression of IFN itself. Among both the infected and uninfected populations, there are many cells with high expression of ISGs and little expression of IFN, but no cells that express high levels of IFN without also substantially expressing ISGs.

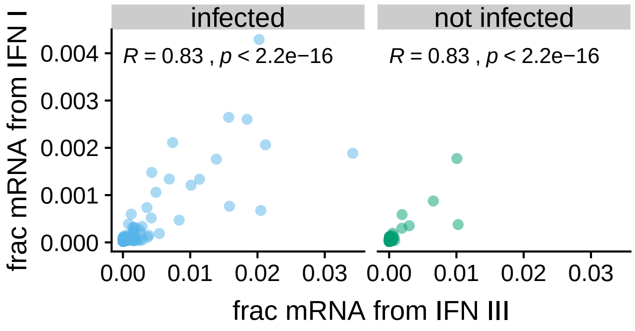

FIG S7 The correlation between the fraction of cellular mRNA derived from type I and type III IFN in the A549 cells in our single-cell transcriptomics. Each point represents one cell. The plots are faceted by whether the cells are called as infected, and the Pearson correlation coefficient is shown. Because type I and type III IFN expression are highly correlated, for the remainder of the paper we group them together and refer to their combined expression as the level of IFN.

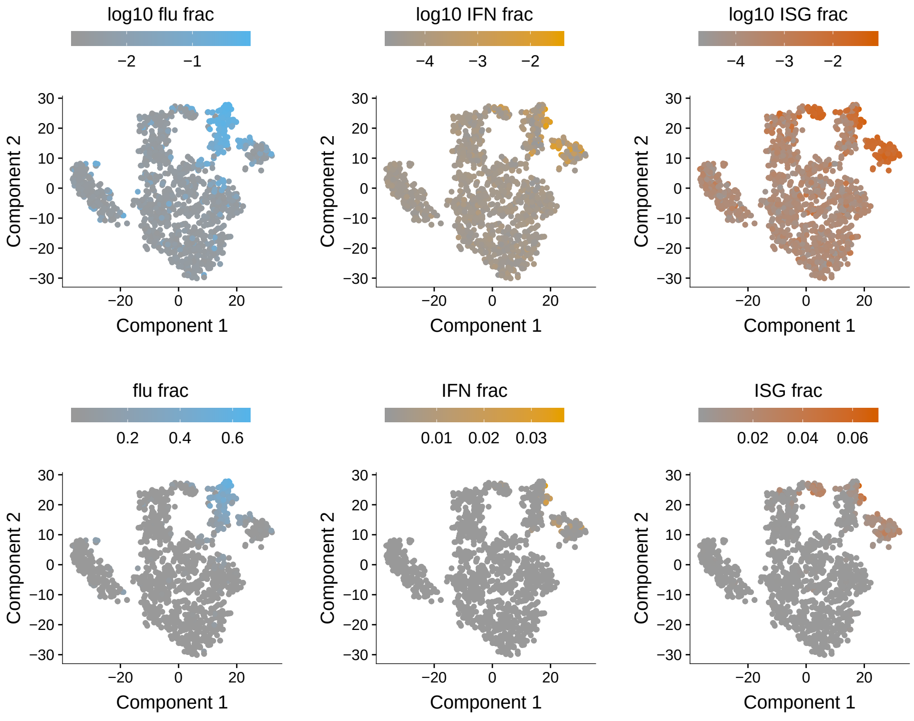

FIG S8 Unsupervised t-SNE clustering shows that cell-to-cell variation in expression of influenza, IFN, and ISG transcripts substantially contributes to the structure of the data. To generate an unbiased representation of the factors that distinguished the transcriptomes of the cells in our experiments, we used unsupervised t-SNE clustering as implemented in Monocle to generate a two-dimensional representation of the data. In the t-SNE plot, each point is a different cell, and cells with similar transcriptomes are closer together. Each panel shows the same t-SNE plot, but the cells are colored differently in each panel based on the amount of viral, IFN, or ISG mRNA, shown on a log (top) or linear (bottom) scale. As is clear from this plot, expression of influenza, IFN, and ISG genes contributes substantially to the structure of the data, since cells with high expression of these genes clearly group together.

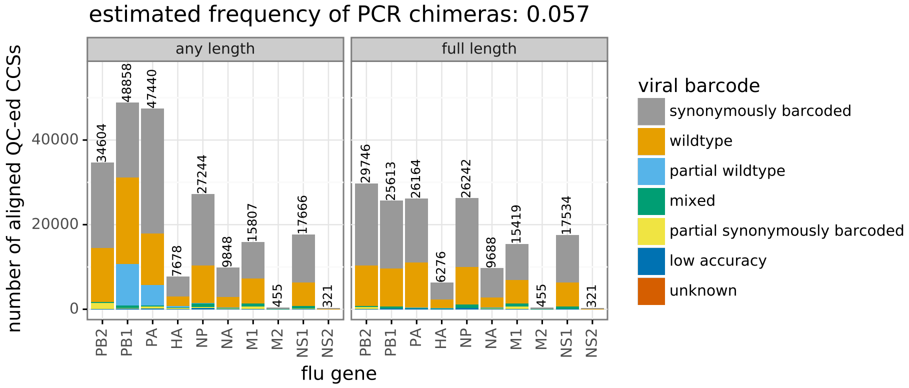

FIG S9 The number of PacBio CCSs that passed quality-control steps and aligned to an influenza virus gene. These sequences were obtained using several PacBio runs, most of which were intentionally loaded with different amounts of the various viral genes to increase coverage on genes that were needed in order to obtain the full sequences of virions infecting cells (see File S7). Because of this unequal loading and the inherently different PCR amplification efficiencies of different viral genes, unlike the transcriptomic data in Fig. 3, the numbers of CCSs for different genes should *not* be taken as an indicator of their abundance in the infected cells. Especially for the polymerase genes (PB2, PB1, and PA), many CCSs corresponded to genes with internal deletions, since these shorter forms of the genes were preferentially amplified during PCR. Therefore, the plot is faceted by the number of CCSs for any length of the gene, and for full-length genes. Note that the disproportionate sequencing of the shorter internally deleted genes does not greatly affect the genotype calling in Fig. 4 since UMIs were used to collapse sequences derived from the same cDNA, and cell barcodes were used to collapse sequences from the same cell. The bars in the plot are colored by whether the sequence is derived from the wild-type viral variant, the synonymously barcoded viral variant, or represents a mixed-barcode molecule (see panel B). From the frequencies of these different forms, we estimate that 5.7% of molecules are chimeric due to PCR strand exchange. About half of these PCR chimeras could be identified by the presence of mixed viral barcodes and removed from subsequent analyses, leaving ~3% un-identified chimeras. For some molecules (mostly polymerase genes with internal deletions) one of the barcode sites was deleted from the molecule and so the barcode identity could only be partially called. A negligible number of molecules have low-accuracy sequence or unexpected nucleotide identities at the sites of the viral barcodes.

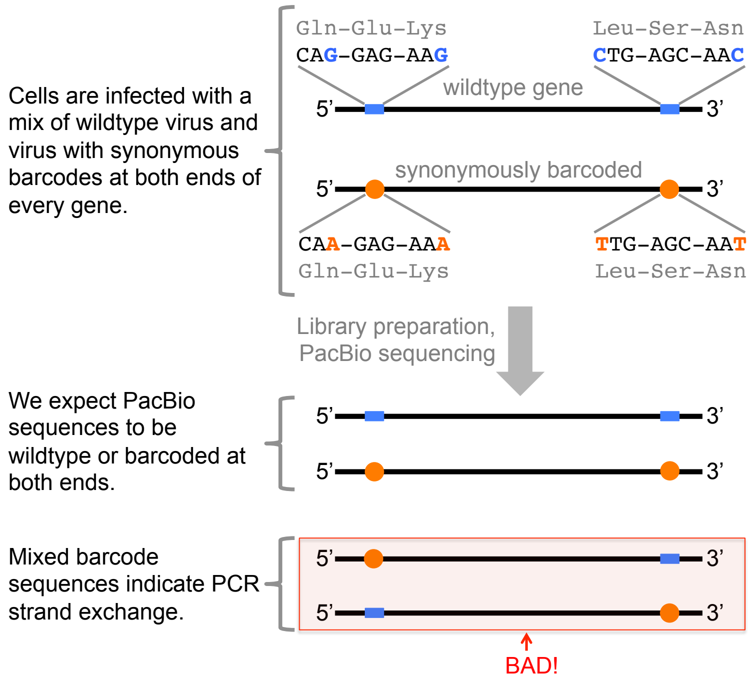

FIG S10 Strategy for detecting strand exchange during sequencing of full-length viral genes. The library preparation for PacBio sequencing of the cDNA for the full-length viral genes required many cycles of PCR. A major concern is that strand exchange during this PCR could scramble mutations and 10X cell barcodes / UMIs from different molecules. We detect PCR strand exchange by leveraging the fact that our cells were infected with a mix of wild-type virus and virus carrying synonymous barcodes near both termini of each gene. If there is no strand exchange, all molecules should either be wild-type or have the synonymous barcoding mutations at *both* termini. Strand exchange will create some molecules that have wild-type nucleotides at one termini and synonymous barcoding mutations at the other termini. Fig. S9 shows the frequencies with which these different types of molecules were observed during the PacBio sequencing. Note that since the rate of homologous recombination in influenza virus in negligible, such mixed-barcode molecules are *not* expected to be generated naturally during co-infection.

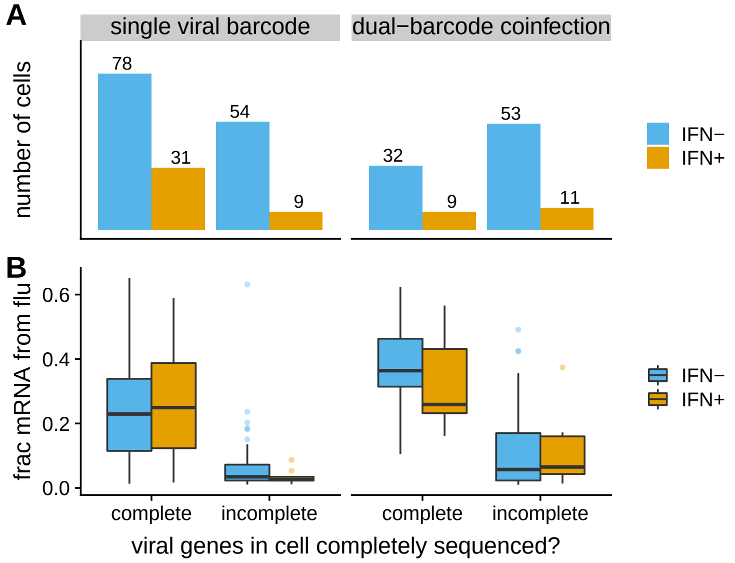

FIG S11 Number of cells for which we could determine the full sequences of all genes expressed by the infecting virion(s). (A) We could call the complete genotypes of the infecting virion(s) for the majority of cells infected with just a single viral barcode variant, but only a minority of cells co-infected with both viral barcodes. (B) The cells for which we could call complete viral genotypes tended to have higher expression of viral mRNA than cells for which we could not call complete genotypes. Both facts make sense. Cells with more viral mRNA are more likely to have their viral cDNA captured in the PacBio sequencing, which is only captures a small fraction of the total transcripts identified by the 3'-end sequencing transcriptomic sequencing. The lower calling rate for dual-barcode co-infections is probably because these co-infections have more viral genes that must be sequenced (potentially a copy of each viral gene from each viral variant), increasing the chances that one of these genes is missed by the PacBio sequencing. An important implication of this plot is that the cells for which we call complete viral genotypes are *not* a random subsampling of all infected cells in the experiment, but are rather enriched for cells that have high levels of viral mRNA and do not have dual-barcode viral infections. Note also that this plot is limited to the cells that were called as infected (Fig. 3C) and could clearly be classified as IFN- or IFN+ (Fig. 3G).

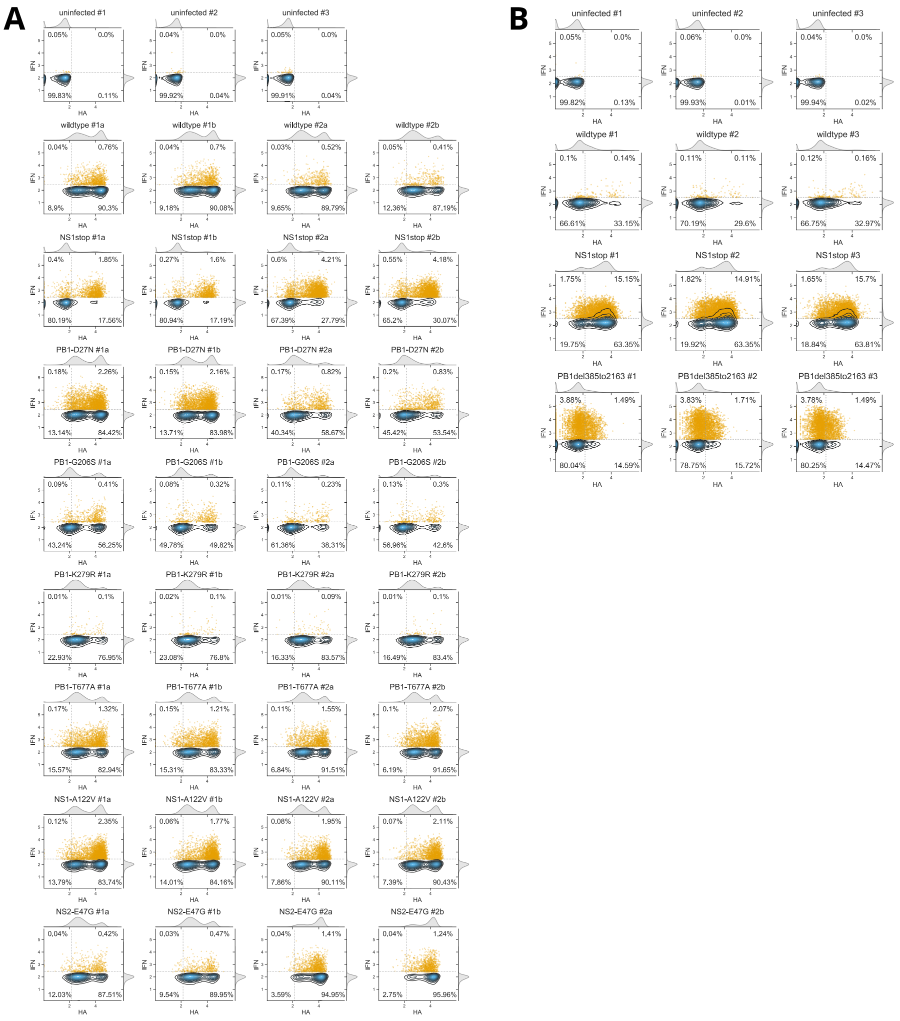

FIG S12 Flow cytometry data for Fig. 6. (A) Data for Fig. 6A. A549 cells with the *IFNL1* reporter driving LNGFRΔC-ZsGreen were infected with stocks of the indicated mutant. After 13 hours, cells were stained for HA protein and analyzed by FACS. Contour plots show density of all cells, and IFN+ cells are also indicated by orange dots. Cells were classified as HA+ or IFN+ based on gates set to put 0.05% of uninfected cells in these populations. For infected cells, the percentage IFN+ was calculated among the HA+ cells (since these are the ones that are infected). For uninfected cells, the percentage IFN+ was calculated among all cells, since uninfected cells do not express HA. For each viral mutant, two independent stocks were assayed in duplicate (i.e., #1a and #1b are one viral stock, and #2a and #2b are the other). The infections with replicate #1 of the wild-type virus were performed at an MOI of 0.1 as determined by TCID50, and all other viruses were infected at an equivalent particle number as determined by HI assay. (B) Data for Fig. 6B. The virus with the deletion in PB1 cannot be normalized by HA expression since it expresses less HA due to the lack of secondary transcription. Therefore, all cells were infected at an equivalent MOI of 0.3 as determined by TCID50 on MDCK-SIAT1 cells for wild type and NS1stop, and on MDCK-SIAT1 cells expressing PB1 for PB1del385to2163. Fig. S13 shows that at these equivalent TCID50s, all variants had similar amounts of transcriptionally active virus in the absence of secondary transcription. The percent IFN+ was calculated for *all* cells (HA+ and HA-) since that is a more fair comparison for PB1del385to2163.

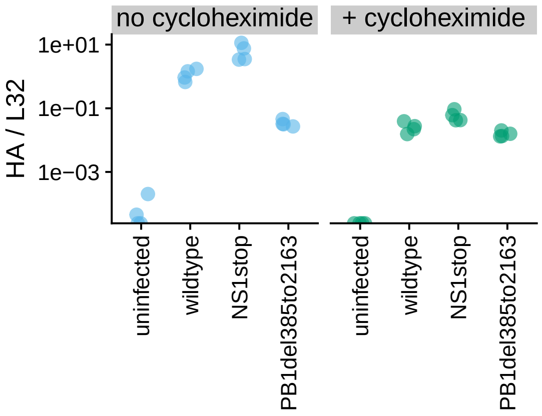

FIG S13 Validation that the infections in Fig. 6B and Fig. S12B were performed at similar doses of virions capable of initiating primary transcription. In this experiment, A549 cells were infected at MOI of 0.4 (based on TCID50 as described in Fig. S12B), and then after 8 hours mRNA was harvested for qPCR on oligo-dT primed reverse transcription products. The y-axis shows the ratio of viral HA mRNA to the housekeeping gene L32. These infections were performed in the presence of absence of 50 μg/ml cycloheximide, which blocks protein synthesis and hence secondary transcription by newly synthesized viral proteins. In the absence of cycloheximide, the viruses with deletions in PB1 produced less viral mRNA presumably because they could not produce PB1 protein for secondary transcription. But in the presence of cycloheximide, all viruses produced similar amounts of viral mRNA, indicating that the dose of particles active for primary transcription is roughly equivalent across variants. Each measurement was performed in quadruplicate.

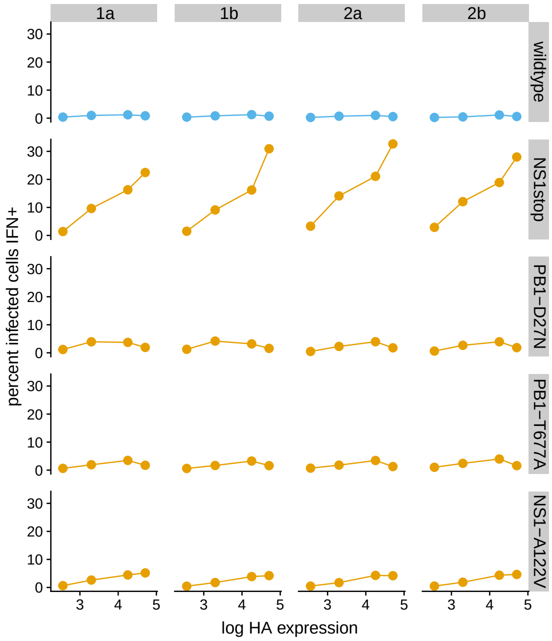

FIG S14 A more detailed version of the data summarized in Fig. 7. For each virus and replicate, we binned the infected cells in Fig. S12 into HA expression quartiles based on the flow cytometry measurements. We then calculated the percent of cells that were IFN+ in each quartile. The plots show the mean HA expression of the quartile versus the precent of cells that are IFN+. The results clearly show that for the NS1stop and to a lesser extent the NS1-A122V variants, more viral protein (higher HA signal) correlates with IFN induction. Fig. 7 summarizes these same data by simply showing the ratio of percent IFN+ between the highest and lowest quartile.

FILE S1 Sequences of the IFN reporters in Fig. 1A are at https://github.com/jbloomlab/IFNsorted_flu_single_cell/tree/master/paper/figures/IFN_stochastic/IFN_reporter/plasmids. This file is also available on Data Dryad Digital Repository at https://doi.org/10.5061/dryad.nh053c6.

FILE S2 Genbank files giving sequences of the wild-type and synonymously barcoded viruses are at https://github.com/jbloomlab/IFNsorted_flu_single_cell/blob/master/data/flu_sequences/flu-wsn.gb and https://github.com/jbloomlab/IFNsorted_flu_single_cell/blob/master/data/flu_sequences/flu-wsn-double-syn.gb. This file is also available on Data Dryad Digital Repository at https://doi.org/10.5061/dryad.nh053c6.

FILE S3 A text file giving the primers used to amplify the influenza cDNAs for PacBio sequencing is at https://github.com/jbloomlab/IFNsorted_flu_single_cell/tree/master/paper/figures/WorkflowSchematic/PacBio_primer_list.txt. This file is also available on Data Dryad Digital Repository at https://doi.org/10.5061/dryad.nh053c6.

FILE S4 A CSV file giving the genotypes in Fig. 4 is at https://github.com/jbloomlab/IFNsorted_flu_single_cell/blob/master/paper/figures/single_cell_figures/genotypes.csv. This file is also available on Data Dryad Digital Repository at https://doi.org/10.5061/dryad.nh053c6.

FILE S5 A CSV file giving the viral mutations and related information in Fig. 5 is at https://github.com/jbloomlab/IFNsorted_flu_single_cell/blob/master/paper/figures/single_cell_figures/mutations.csv. This file is also available on Data Dryad Digital Repository at https://doi.org/10.5061/dryad.nh053c6.

FILE S6 Genbank plasmid maps for the mutant genes cloned into the pHW* bidirectional reverse genetics plasmid are at https://github.com/jbloomlab/IFNsorted_flu_single_cell/tree/master/paper/figures/FluVariantPlasmidMaps. This file is also available on Data Dryad Digital Repository at https://doi.org/10.5061/dryad.nh053c6.

FILE S7 The Jupyter notebook that analyzes the PacBio data is at https://github.com/jbloomlab/IFNsorted_flu_single_cell/blob/master/pacbio_analysis.ipynb. This file is also available on Data Dryad Digital Repository at https://doi.org/10.5061/dryad.nh053c6.

FILE S8 The Jupyter notebook that analyzes the annotated cell-gene matrix is at https://github.com/jbloomlab/IFNsorted_flu_single_cell/blob/master/monocle_analysis.ipynb. This file is also available on Data Dryad Digital Repository at https://doi.org/10.5061/dryad.nh053c6.
